# Lifelong behavioral screen reveals an architecture of vertebrate aging

**DOI:** 10.1126/science.aea9795

**Published:** 2026-03-12

**Authors:** Claire N. Bedbrook, Ravi D. Nath, Libby Zhang, Scott W. Linderman, Anne Brunet, Karl Deisseroth

**Affiliations:** 1 Department of Bioengineering, Stanford University, Stanford, CA, USA.; 2 Department of Genetics, Stanford University, Stanford, CA, USA.; 3 Department of Electrical Engineering, Stanford University, Stanford, CA, USA.; 4 Wu Tsai Neurosciences Institute, Stanford University, Stanford, CA, USA.; 5 Department of Statistics, Stanford University, Stanford, CA, USA.; 6 The Phil and Penny Knight Initiative for Brain Resilience at the Wu Tsai Neurosciences Institute, Stanford University, Stanford, CA, USA.; 7 Glenn Center for the Biology of Aging, Stanford University, Stanford, CA, USA.; 8 Department of Psychiatry and Behavioral Sciences, Stanford University, Stanford, CA, USA.; 9 Howard Hughes Medical Institute, Stanford University, Stanford, CA, USA.

## Abstract

**INTRODUCTION:**

With increased age comes a substantially increased risk of devastating illnesses, including cancers, cardiovascular diseases, and dementias. As elderly populations grow, so does the urgency to better understand the lifelong process of aging. We hypothesized that fundamental insights into the dynamic process of aging could come from continuously observing individuals across life until aging-related death. However, given the long timescale of vertebrate aging, continuous observation across an individual vertebrate animal’s life has not been practical.

**RATIONALE:**

We studied the African turquoise killifish, a vertebrate model for aging with a naturally compressed lifespan (median 4 to 8 months), to continuously record behavior from puberty to death and explore the architecture of adult lifespan progression and aging. Behavior is a rich readout of animal state that integrates diverse features of multiorgan system physiology, including the core nervous system functions of sensation, cognition, and action. We designed an unbiased screen to systematically explore how behavioral patterns change with age and to determine whether behavior could predict future aging differences and even remaining lifespan. This unbiased approach also allowed us to explore the impact of interventions on behavior and to test for the presence of behavioral stages that define progression through adult life.

**RESULTS:**

We built a system to continuously screen behavior across the entire adult lifespan of individual animals, which enabled the initial investigation of vertebrate multidimensional behavioral dynamics spanning timescales from single video frames (tens of milliseconds) to whole lifespans (~250 days from puberty to death) in a systematic and quantitative manner. With this view into the process of aging, we discovered that behavioral trajectories of short-lived animals are distinct from those of long-lived animals. Based on these behavioral trajectories, we separated chronologically age-matched animals predicted to be long- versus short-lived and performed multiorgan transcriptomic profiling. Animals destined for a long lifespan exhibited transcriptomic changes in ribosomal and metabolic pathways but not in other key aging-related pathways such as inflammation. Through our machine-learning models, we found that the behavior of an individual at a relatively young age sufficed to predict future short or long lifespan, and key predictive behaviors appeared to be conserved across the animal kingdom. The noninvasive behavioral screen also enabled the exploration of how a human-relevant longevity intervention (dietary restriction) influences the process of aging. Finally, our continuous tracking of the aging process from adolescence to death revealed the surprising observation that animals exhibit notable transitions between stereotyped behavioral stages at distinct ages. These data suggest a model for the architecture of adult life progression, in which the process of aging encompasses the succession of discrete life stages, rather than a gradual continuous decline.

**CONCLUSION:**

We designed and built a platform to continuously track naturalistic behavior across lifespan from adolescence to death, allowing for a high-resolution unbiased screen of the process of aging in vertebrates. We found that animal behavior can be a highly informative noninvasive readout of the process of aging and that vertebrate animals progress through adulthood in an orderly sequence of stable and stereotyped behavioral stages. The lifespan architecture described here advances basic understanding of biological aging and may also enable targeted mechanistic and therapeutic discovery work that is relevant to human aging and age-related disease.

## Abstract (summary)

Mapping behavior of individual vertebrate animals across lifespan could provide an unprecedented view into the lifelong process of aging. We created a platform for high-resolution continuous behavioral tracking of the African killifish across natural lifespan from adolescence to death. We found that animals follow distinct individual aging trajectories. The behaviors of long-lived animals differed markedly from those of short-lived animals, even relatively early in life, and were linked to organ-specific transcriptomic shifts. Machine-learning models accurately inferred age and even forecasted an individual’s future lifespan, given only behavior at a young age. Finally, we found that animals progressed through adulthood in a sequence of stable and stereotyped behavioral stages with abrupt transitions, revealing precise structure for an architecture of aging.

## Editor’s summary

Continuous recording of a vertebrate’s adult life from adolescence until death would provide a complete view into the behavioral architecture of aging. However, the long time scale and complexity of vertebrate aging have so far precluded such observations. Bedbrook *et al*. leveraged major advances in machine learning and computer vision to follow nearly every moment of the adult life of a naturally short-lived vertebrate, the African killifish. The authors found that individual animals followed distinct aging trajectories defined by abrupt transitions in behavior. These results might lead to better understanding of the aging process in other vertebrates, including humans. —Mattia Maroso

## Lifelong behavioral screen.

To investigate the lifelong progression of aging, we continuously tracked behavior from adolescence to death in the naturally short-lived African turquoise killifish. Naturalistic behavioral readout, which is noninvasive, revealed individual animal aging trajectories across life. Behavior not only can be used to infer age but also can forecast future lifespan. The continuous nature of behavioral tracking suggests an architecture of aging whereby individuals progress through adulthood in an orderly sequence of stable and stereotyped behavioral stages.

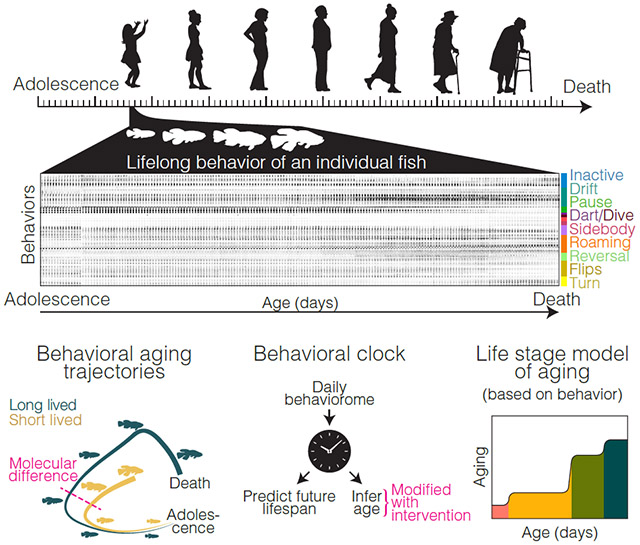

Deep insight into the processes of life has come from the dynamic observation of intact biological systems over long timescales. For example, continuous inspection of developing embryos has provided critical insight into the processes governing the transition from a single cell to a fully formed organism (1-7). However, these dynamical maps have typically ended with completion of initial organismal development. Fundamentally distinct classes of insight might be derived from continuing to observe individual organisms across the entirety of adulthood all the way to aging-related death. Continuous tracking during aging could reveal new information regarding unrecognized postmaturity life stages, as well as natural health decline during aging, especially as age is the primary risk factor for most chronic diseases (8-10). However, given the long timescale of vertebrate aging, such continuous observation across an individual vertebrate animal’s life has not been practical.

Behavior is a rich and diverse readout of animal state (11-15)—integrating diverse features of sensation, cognition, and action—which has been shown to reflect the aging process in worms (16-20), flies (21, 22), mice (23, 24), and humans (25-28). Recent advances in computer vision and animal-tracking technology (14, 29-33) have enabled the continuous recording and quantification of naturalistic, self-motivated behaviors. Yet the required timescale for widely applied vertebrate model systems [for example, a 2- to 3-year median lifespan in mice and an ~3-year median lifespan in zebrafish (9)] is prohibitively long for whole-lifespan, continuous recording of behavior. Here, we leveraged the African turquoise killifish, a vertebrate model for aging with a naturally compressed lifespan (median 4 to 8 months) (34-49), to continuously record behavior from puberty to death and explore the architecture of adult development and aging. With this lifelong behavioral screen, we sought to systematically determine how naturalistic behaviors change with age and whether behavior was sufficient to predict aging differences and even remaining life. Our unbiased approach also allowed us to explore the impact of interventions on behavior and to uncover discrete behavioral stages throughout adult life.

## System for continuous behavioral tracking across life

**Fig. 1.**
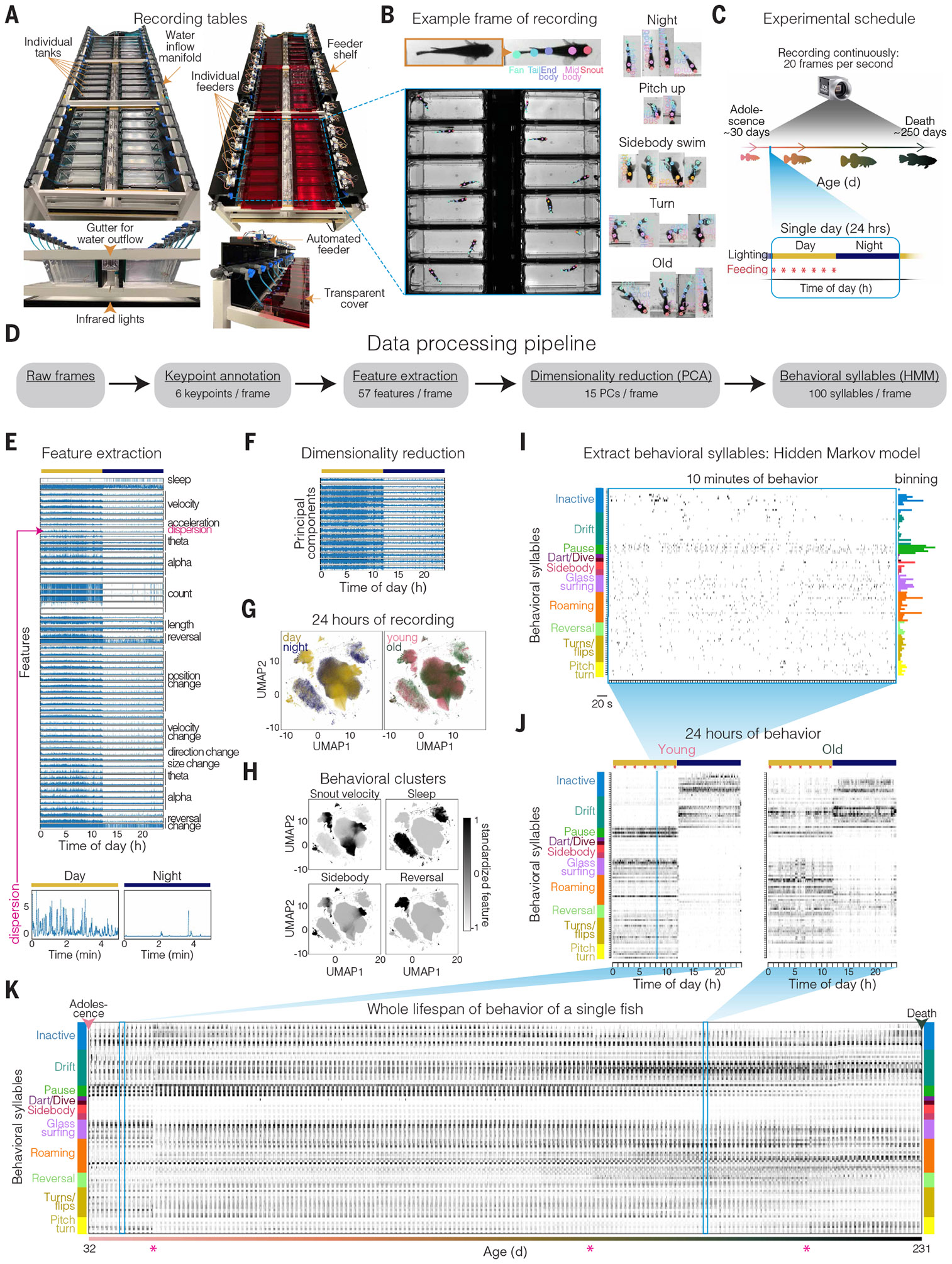
The recording system enables continuous behavioral tracking from adolescence to death. (**A**) Long-term recording setup: 2.8-liter tanks fixed in a custom table rack with continuous water inflow and outflow. Automated feeders were fixed above each individual tank on a feeder shelf. An infrared-transparent red acrylic lid was placed above all tanks, with an infrared backlight placed below the tanks. (**B**) Example frame from the Basler industrial camera (capturing 20 frames per second) imaging a single recording table. Six key points (snout, midbody, endbody, tail, fan, and sidebody) were predicted from a trained deep convolutional neural network overlayed on an example frame. Cropped individual frames of a recording show animals at different times of day, age, and positions. (**C**) Experimental schedule for recordings from adolescence to death with a 12-hour–12-hour light-dark cycle. Yellow indicates day, and dark blue indicates night. (**D**) Data-processing pipeline from raw video frames to behavioral syllable usage. (**E**) Pose features extracted from key-point data for a single animal in 1 day (24-hour cycle) of its life. The inset below shows a zoom-in of a 5-min interval during the day and night, highlighting dispersion. (**F**) PCA of pose features for the same animal and day from data shown in (E). (**G**) UMAP embedding of pose feature PCs of data from a single day for nine different fish down-sampled to show one every five frames, with each point being a single frame of recording and color maps indicating day-versus-night frames or young (45 days)–versus–old (270 days) frames. (**H**) UMAP embedding of pose feature PCs with a heatmap for four select pose features. (**I**) Sampling of 100 HMM-derived behavioral syllables for a single fish during a 10-min interval during 1 day of its life. Behavioral syllables are ordered according to similarity (D_KL_) and hierarchical clustering of distinct types of behavior shown in color along the left (see fig. S4B for clustering). The histogram shows time spent in each behavioral syllable during the 10-min bin on the right. (**J**) Behavioral syllable usage across 24 hours for a single animal when young (40 days) versus across 24 hours for the same animal when old (180 days). (**K**) Whole-lifespan behavioral syllable usage for a single animal from 32 days old until death at 231 days, with a pink asterisk highlighting transitions in syllable use (see Fig. 6 for analysis of these transitions across the population).

To continuously measure behavior of individuals in a highly scalable manner, we designed a system to house and track many individual animals in a physiologically suitable, freely moving environment over the entire adult lifespan (Fig. 1 and fig. S1). We began with male African turquoise killifish individually housed with circulating water flow (Fig. 1A). The animals received seven feedings each day at fixed times using custom programmable automated feeders (50). Cameras were mounted overhead and ran continuously at 20 frames per second (Fig. 1B and movie S1). Each animal was placed into its recording tank at puberty (sexual maturity, 3 to 4 weeks of age), and all behaviors were tracked continuously until death (Fig. 1C).

To track animal movement, we trained a deep convolutional neural network that predicts six key points along the body in all frames of the whole-lifespan recording (Fig. 1B and movie S2; see Materials and methods). From tracked key points for each animal, we calculated “pose features” (a total of 57; Fig. 1D and data S1; see Materials and methods), which describe parameters such as velocity, body curvature, and swim direction (Fig. 1E). We then used principal components analysis (PCA) to represent pose feature dynamics with fewer dimensions [57 pose features to 15 principal components (PCs) with >80% variance explained; Fig. 1F and fig. S2A]. Pose features over a 24-hour interval revealed distinct behavioral clusters (Fig. 1, G and H, and figs. S2B and S3). Different behavioral clusters were used in day versus night (linear separability mean cross-validation accuracy = 0.71) (Fig. 1G and fig. S2C). By contrast, young (45 days old) and aged (270 days old) animals showed a large degree of overlap in behavioral cluster usage (linear separability mean cross-validation accuracy = 0.56) (Fig. 1G and figs. S2D and S3), indicating that aging did not completely restructure the behavioral repertoire.

To investigate how the behavioral repertoire did change across lifespan in individual animals, we extracted behavioral motifs. We took an unsupervised approach using a Gaussian hidden Markov model (HMM) to model stereotyped subsecond behavioral motifs from the pose feature dynamics, referred to as “behavioral syllables” (Fig. 1, I to K). HMM-derived behavioral syllables (11, 13, 51) have been shown to reflect animal internal states (52-54). Owing to the huge amount of data, we developed a distributed, stochastic expectation-maximization procedure to fit the HMM to >3 × 10^8^ frames of data (fig. S4A; see Materials and methods), resulting in 100 behavioral syllables, including active behavioral syllables (for example, flips, bursts, and reversals) as well as inactive behavioral syllables (for example, slow drifting and resting). Over 24 hours, animals sampled distinct behavioral syllables during the day and night (Fig. 1J and fig. S5A). We observed a clear shift in behavioral syllable usage for individual animals at young (40 days old) versus aged (180 days old) time points (Fig. 1J). Continuous sampling of behavior across the whole adult lifespan of individual animals enabled the first investigation of vertebrate multidimensional behavioral dynamics across timescales from frames (milliseconds) to whole lifespans (~250 days from puberty to death) in a systematic and quantitative manner (Fig. 1K and fig. S6).

## Global behavioral changes during aging from whole-lifespan tracking

**Fig. 2.**
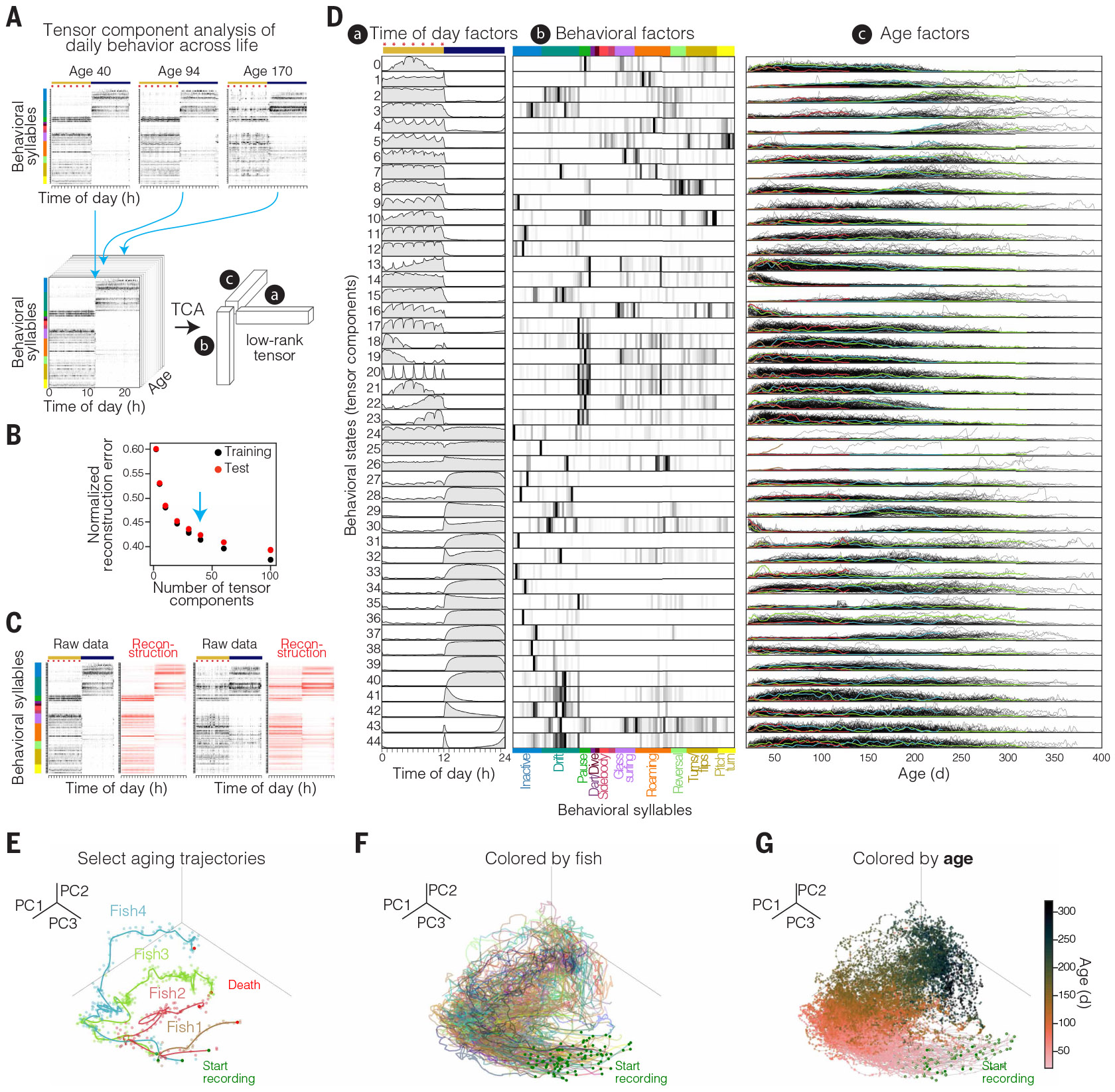
Global behavior changes with age reveal distinct aging trajectories. (**A**) Schematic of TCA setup to investigate whole-lifespan behavioral syllable use. Red asterisks indicate feeding times, the light-dark cycle is indicated by a yellow–dark-blue bar, and behavioral syllables are ordered and clustered as in Fig. 1I. (**B**) Normalized reconstruction error for both training and held-out test-set data for models trained with different numbers of TCs. (**C**) Comparison of raw versus TCA reconstructed syllable data for two randomly selected days. (**D**) Trained 45-TC model showing time-of-day factors, behavioral syllable factors, and age factors for each TC (behavioral state). Age factors show weighting of each factor over age, with each thin line representing the trajectory of a single animal. Select animals shown in color correspond to animals shown in (E). (**E**) Top three PCs of the 45-TC data used to visualize aging trajectories of select individual animals from the start of recording (green circle) to death (red circle). Each animal is shown in a different color. Points show individual days, and lines show smoothed trajectories. (**F** and **G**) Aging trajectories of >100 animals, with each animal in a different color (F) and with each point colored by animal age (G).

To identify behavioral changes during aging that were consistent across many individual animals, we developed an approach to address the challenge of massive dimensionality of the dataset. For each animal on each day, there are 1.728 × 10^6^ frames of recording with one of 100 possible behavioral syllables per frame, and for lifespan recordings, 81 animals were recorded for a mean of 220 days (range 23 to 371 days). To represent daily behaviors by fewer dimensions while preserving the strong circadian structure within the dataset, we used tensor component analysis (TCA) (55), which is an unsupervised method capable of extracting low-dimensional factors from multidimensional data. We trained non-negative TCA models on the whole-lifespan time series restructured into daily behavioral syllable usage (binned at 10-min resolution) concatenating animals across the time dimension such that a single TCA model was trained for all fish (Fig. 2A). Increasing the number of tensor components (TCs) improves reconstruction error, but improvement becomes marginal by ~45 TCs for the held-out test set (~40% unexplained variance; Fig. 2B), and reconstructions built using a 45-TC model strongly resemble raw data (Fig. 2C); we therefore used a model with 45 TCs for next-stage analysis.

TCA approximates the data as a sum of outer products of three vectors: in our case, (a) time-of-day factors, (b) behavioral-syllable factors, and (c) age factors, as shown in Fig. 2, A and D. The time-of-day factors recapitulate the expected circadian dynamics, including distinct TCs dominant during the day (for example, TC1) or night (for example, TC29), as well as TCs that peak at each of the seven feedings (for example, TC20) (subpanel a in Fig. 2D). This analysis groups behavioral syllables that are commonly used together with specific temporal dynamics throughout the circadian cycle to build more complex behavioral states, providing a structure for comparison across ages. Indeed, the amplitude or weighting of each time-of-day and behavioral-syllable factor differs each day and for each animal and is represented as the third factor, the “age factor” (subpanel c in Fig. 2D). Whereas many TCs showed changes with age (for example, TC14), others showed more uniform use across age (for example, TC44), and yet other TCs (for example, TC24) showed large increases in use with age but in only a small subset of animals (subpanel c in Fig. 2D). This TCA analysis thus represents a robust way to reduce data dimensionality, enabling both qualitative and quantitative comparisons between behavioral states across lifespan.

## Distinct aging trajectories based on behavior

Do individuals exhibit distinct behavioral aging trajectories? To investigate this, we visualized age factors across life for each animal. We performed PCA on all 45 TCs identified above and plotted the top three PCs (Fig. 2, E to G) (PC1 to PC3: 40% explained variance; fig. S7). Focusing on the aging trajectories of select fish illustrates that individual animals exhibited distinct aging trajectories in behavioral space (Fig. 2E), suggesting that individuals are not all aging in the same way. The behavioral trajectories were not linear: Animals spent more days in some parts of behavioral space than others (Fig. 2E and figs. S8 and S9). Across large cohorts of animals, young animals generally started in a similar place (low variance across 45 TCs; fig. S10A), but we observed that aging animals fanned out into distinct behavioral trajectories (Fig. 2F) (increasing variance with age across all 45 TCs; fig. S10A), and the position along these behavioral trajectories was strongly correlated with animal age (Fig. 2G). We obtained similar results using uniform manifold approximation and projection (UMAP) embedding for visualization instead of restricting observations to the top three PC dimensions (fig. S10, B and C).

**Fig. 3.**
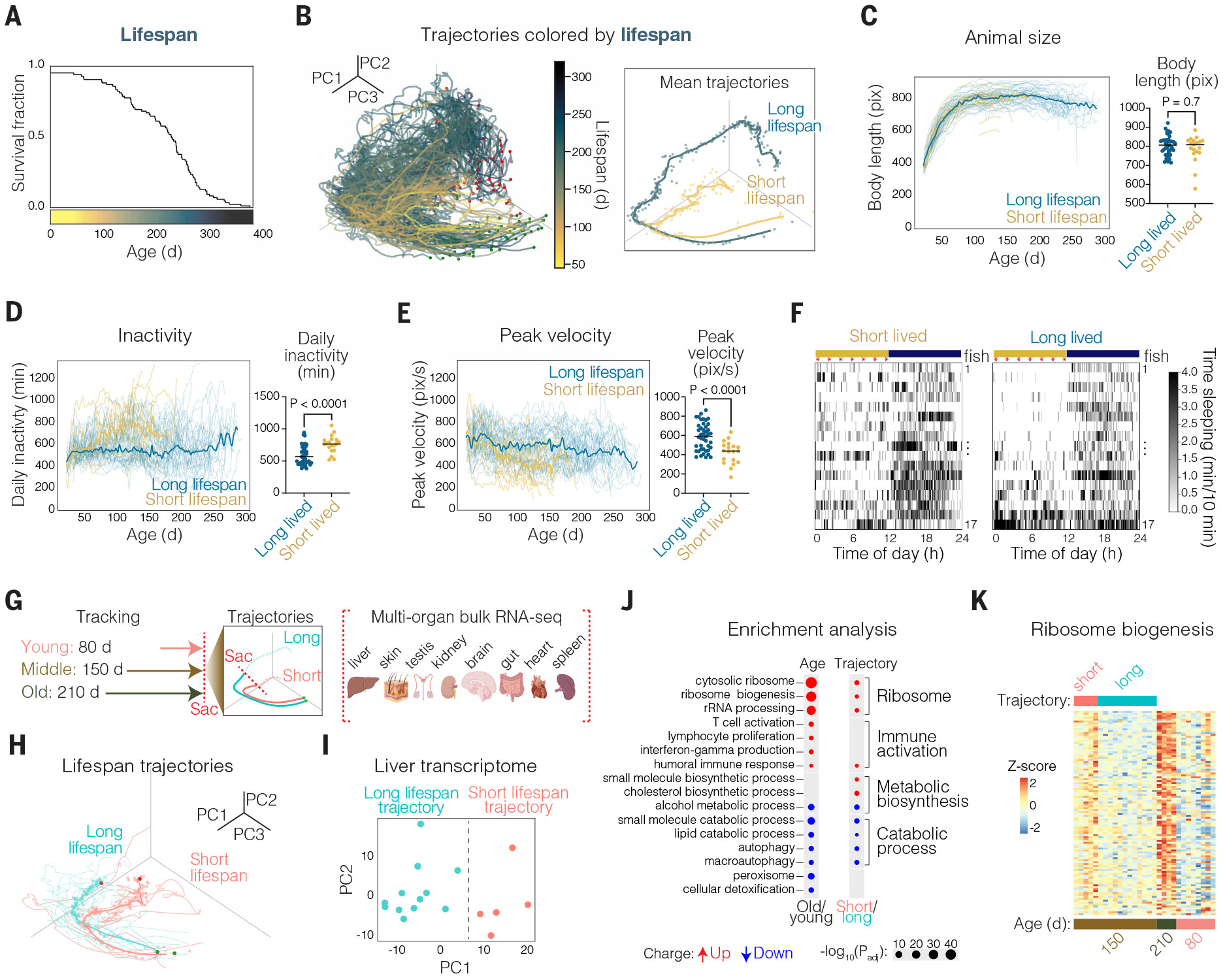
Behavioral aging trajectories diverge early in life based on future lifespan and are linked to large transcriptomic changes in the liver. (**A**) Kaplan-Meier lifespan curve of tracked animals (*n* = 81). (**B**) Aging trajectories of 81 animals colored by future lifespan. The inset shows the mean trajectory of distinct lifespan groups: long lifespan (≥200 days) and short lifespan (<200 days and ≥120 days to exclude the impact of individuals that died prematurely). (**C** to **E**) Animal size (length) (C), inactivity (D), and peak velocity (E) across life (left) and at 100 days old (right) grouped by lifespan. Mann-Whitney *U* test was used to determine significance. At 100 days old, there is a significant positive correlation between peak velocity and future lifespan (Spearman’s correlation: *P* = 0.005, ρ = 0.35). pix, pixels. (**F**) Heatmap of time spent sleeping across 24 hours at 100 days old, with each row representing a different animal and animals grouped by lifespan. Red asterisks indicate feeding times, and the light-dark cycle is indicated by a yellow–dark-blue bar. Seventeen animals were randomly sampled from the long-lived group to match the size of the short-lived group. (**G**) Scheme for organ RNA sequencing experiment of animals tracked to different age endpoints: young (80 days; *n* = 8), middle-aged (150 days; *n* = 17), and old (≥210 days, *n* = 4). At each endpoint, organs were harvested for bulk RNA sequencing. Sac, sacrifice. (**H**) Behavioral aging trajectories of 17 animals colored by inferred future-lifespan trajectory. (**I**) PCA of liver transcriptome for each animal in the middle-aged group (150 days; *n* = 17) colored by future-lifespan trajectory based on behavior. (**J**) GO enrichment analysis of the liver transcriptome as a function of age (old versus young) and lifespan trajectories based on behavior (short- versus long-lifespan trajectory) highlighting significant GO terms. (**K**) Heatmap of genes involved in the ribosome biogenesis GO term. Each row is a gene, and each column is an animal. The color bar below the heatmap indicates the age group of each individual animal, and the color bar above shows the lifespan trajectory of the middle-aged (150 days) group.

Given that behavior is a noninvasive and nondestructive readout, we recorded across the complete adult lifespan of each individual. There was variability in lifespan, from short (46 days) to long (394 days) (Fig. 3A), as is typical of all species, even in genetically identical populations (56). Marking trajectories by the lifespan of each fish revealed that animals with a long lifespan (blue in Fig. 3B) exhibited a distinct trajectory from that of animals with a short lifespan (yellow in Fig. 3B). Furthermore, comparing the mean trajectories of animals with long versus short lifespan revealed that long-lived (≥200 days) and short-lived (≥120 days and <200 days) trajectories started together in youth but diverged quite early in life, with a branch point at ~70 days old (Fig. 3B). This branching took place when animals were still relatively young and a long time before death, even for the short-lived population (50 to 130 days before death), suggesting that there are physiological changes relatively early in life that dictate an animal’s aging trajectory and future lifespan, and similarly for health span (fig. S11).

What is different between long- versus short-lived animals? There were not large differences in animal size (length) (Fig. 3C), growth rate, or feeding behavior (TC 20 in Fig. 2D; see fig. S12B) in the long- versus short-lifespan groups across life; moreover, both long- and short-lived animals spent a similar proportion of their life before death with reduced food consumption, resulting in residual uneaten food (fig. S12A). Thus, multiple quantitative measures revealed no evidence for stark differences in food consumption between the two groups. Long- and short-lived animals also showed similar peak and median velocity at young ages (<70 days). However, by 100 days, long-lived animals spent significantly more time active than short-lived animals (*P* < 0.0001 Mann-Whitney *U* test) and exhibited significantly higher peak velocity (“sprinting”) and median velocity (peak velocity, *P* < 0.0001; median velocity, *P* < 0.0001 Mann-Whitney *U* test) (Fig. 3, D and E, and fig. S12D). Consistent with this finding, long-lived animals maintained more elevated vigorous darting behavior levels than short-lived animals by 100 days (*P* < 0.0003 Mann-Whitney *U* test; fig. S12C). In addition to these differences in activity and velocity, we also observed stark differences in the circadian timing of active and inactive behaviors when comparing the long- and short-lived groups at 100 days (Fig. 3F and fig. S12F). Specifically, short-lived animals showed increased sleep behavior during the light (day) period, whereas sleep behavior was more restricted to the dark (night) period in the long-lived group at age-matched time points. Thus, before middle age, animals destined for a long lifespan (although the same size) were more active, had higher peak velocity, and showed tighter circadian timing of sleep and active behaviors.

## Molecular differences between long- and short-lived aging trajectories

The continuous behavioral tracking system was designed to noninvasively evaluate the aging trajectory of animals while they are still relatively young, allowing a comparison of animals destined for a long lifespan to those destined for a short lifespan even while early in life. We tracked a cohort of animals from adolescence to middle age (150 days old; *n* = 17 animals), at which point we could reliably distinguish long- versus short-lifespan trajectories based on behavior for each individual (Fig. 3, G and H). We then sacrificed animals and performed bulk RNA sequencing of eight different organs (brain, gut, heart, kidney, liver, skin, spleen, and testis) of each individual to investigate molecular signatures of distinct aging trajectories. For context, in addition to evaluating animals on a long- versus short-lifespan trajectory, we also compared molecular signatures of young versus old animals. For this measurement, we tracked a cohort of animals up to a relatively young age (80 days; *n* = 8) and a cohort of animals up to advanced age (≥210 days; *n* = 4) (Fig. 3G); both groups were sacrificed, followed by bulk RNA sequencing of the eight different organs.

Across multiple organs, PCA could separate the transcriptomes of individuals of the same chronological age but of different lifespan trajectories based on behavior (fig. S13, A and B). The liver had the clearest separation of individual transcriptomes associated with long- or short-lifespan trajectory based on behavior (Fig. 3I), as quantified by Silhouette score (fig. S13B). Unbiased k-means clustering of the liver transcriptomes resulted in two clusters that matched the short- and long-lifespan trajectory groups (fig. S13C), corroborating liver transcriptome separation. This result was intriguing given the liver’s key role in metabolism and sensitivity to interventions that affect longevity (57-59), suggesting possible metabolic underpinnings for the differences in behavioral activity and vigor of long- versus short-lived animals.

Gene set enrichment analysis comparing the liver transcriptomes of individuals on long- versus short-lifespan trajectories revealed major differences in gene ontology (GO) terms relating to ribosome biogenesis, ribosomal RNA (rRNA) processing, translation, and DNA replication, which were all up-regulated in animals on a short-lifespan trajectory (Fig. 3J, fig. S14B, and data S2). These same terms were also all found to be up-regulated with age (Fig. 3J and fig. S14B). By contrast, several age-related changes were not different in animals on short- versus long-lifespan trajectories. For example, we also observed a robust increase with age in GO terms relating to immune activation (T cell activation, lymphocyte proliferation, interferon-γ production) (Fig. 3J), but this result was not seen when comparing animals on short- versus long-lifespan trajectories. Thus, immune or inflammation changes may not causally underlie the differences in lifespan that we observed but rather represent a phenotype associated with natural aging. By contrast, ribosome biogenesis up-regulation (as well as down-regulation of catabolic processes such as autophagy; Fig. 3J), which was seen both in old animals and in animals destined for short lifespan based on behavior (Fig. 3K), could represent candidate molecular drivers of longevity.

## Behavior suffices to infer age

**Fig. 4.**
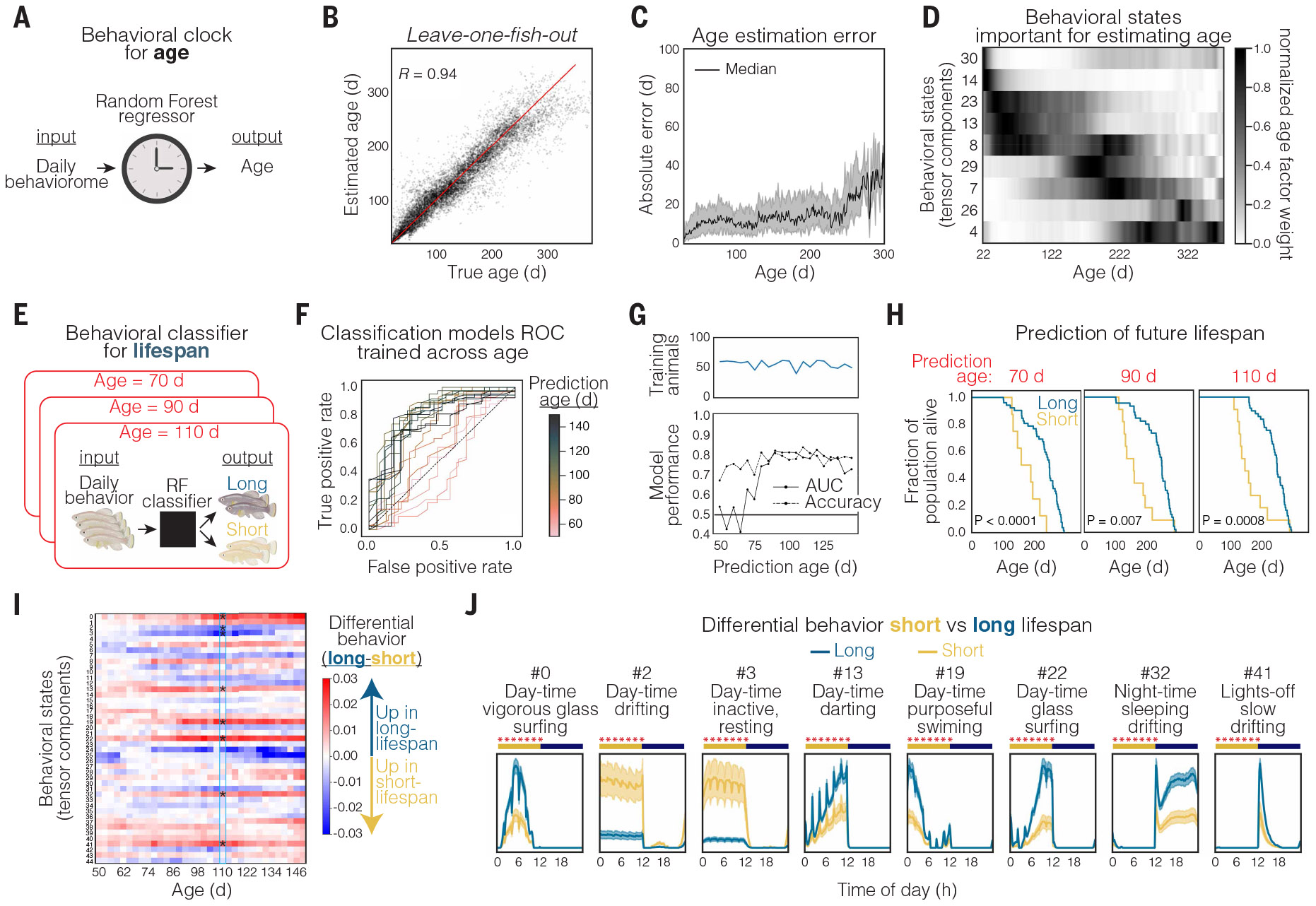
Behavior is highly predictive of age and future lifespan. (**A**) Behavioral clock for age—random forest regression models trained on the 45-dimensional age factors that summarize a fish’s behavior for a given day (that is, the daily “behaviorome”) to estimate age. (**B**) Leave-one-fish-out model estimated age versus true age. (**C**) Leave-one-fish-out model age-estimation absolute error calculated across age. (**D**) Age weighting of the daily behavioral states (TCs) that are most important for model age estimation (based on mean decrease in impurity). (**E**) Behavioral classifier for lifespan—random forest (RF) classifiers trained on behavioral TC data at distinct age windows to classify animals as either short- or long-lived. (**F**) ROC curve for random forest classification models trained on behavioral data from animals at different ages. The coloring of curves is based on the age of the animals used for predictions. (**G**) Shown at the top is the number of animals included in model training across different age groups. Shown at the bottom is classification model performance in both area under the ROC curve (AUC) and accuracy across different age groups. (**H**) Kaplan-Meier lifespan curve of animals predicted to be short-lived (yellow) and long-lived (blue) at various prediction ages (log-rank test). (**I**) Differential behavior of long-lived minus short-lived animals, highlighting specific behavioral states that are significantly different between short- and long-lived groups at the age of 110 days (Mann-Whitney *U* test with Bonferroni correction). (**J**) Highlighting behavioral states (TCs) that are significantly different between short- and long-lived groups at 110 days, plotting mean age-factor weights by time-of-day factor for the short-lived (yellow) versus long-lived (blue) groups (TC numbering as in Fig. 2D).

We asked whether behavioral measures could suffice to estimate the age of an individual, which would be a key step for both quantifying aging and predicting the effect of interventions (60-62), especially because behavior is a noninvasive readout that is well suited for work across the lifespan. To address this question, we built a behavioral clock—a statistical model that takes as its input the 45-dimensional age factors (subpanel c in Fig. 2D) that summarize a fish’s behavior for a given day (the daily “behaviorome”) in an animal’s life and estimates the age of the animal on that day (Fig. 4A). We trained a random forest regressor and evaluated model performance using leave-one-fish-out cross-validation. The age estimated by this model was found to correlate strongly with true age [Pearson correlation coefficient (*R*) = 0.94; Fig. 4B], with a median absolute error (MAE) of only 12 days across ages. At very old ages (>250 days old) the MAE increased to >20 days, likely because of the large drop-off in the amount of training data at these extreme ages (Fig. 4C). We observed consistent results when evaluating model performance on a held-out test cohort of animals (*R* = 0.92, MAE 12 days across ages; fig. S15). These results indicate that the behavior of an individual from noninvasive recording is sufficient to closely estimate animal age across lifespan.

The daily behavioral states that were most important for model estimation were observed to change as a function of age (Fig. 4D). Specifically, the behavioral states that were most important to estimate age (based on mean decrease in impurity; see Materials and methods) at each stage of life were (i) day-time vigorous swimming and exploring behavior in young animals, (ii) night-time slow drifting and sleep behavior in middle-aged animals, (iii) constant (noncircadian) slow drifting turns in old animals, and (iv) drifting backward and reversal flips in very old (geriatric) animals. These waves of distinct behavioral state usage at specific age ranges reveal that aging in adult animals may not be well described as a gradual decline but rather as progression through discrete stages in which distinct combinations of behaviors are used.

## Future-lifespan forecasting based on behavior

We next asked whether daily behavioral measures could predict an animal’s future lifespan. Specifically, would it be possible to forecast, based solely on behavior at a young age, whether animals were destined for short versus long lifespans? To test this possibility, we built classification models at different fixed age ranges. These models input the 45-dimensional age factors (subpanel c in Fig. 2D) that summarize a fish’s behavior for a given day (the daily behaviorome) within a specific age window and then classify the animal as short-lived (<200-day lifespan) or long-lived (Fig. 4E). Whereas classifier performance to predict future lifespan was found to be low at very young ages (<70 days), marked improvement was observed after 70 days (young adults) [receiver operating characteristic (ROC) curve and accuracy >0.7; Fig. 4, F and G].

We used the age-fixed classifiers to classify each animal within a cohort as short- versus long-lived (leave-one-fish-out predictions) and then plotted the true survival curves for the separate groups. This analysis revealed a large separation in lifespan curves (Fig. 4H). Future lifespan predictions were accurate using behaviors at ages 70, 90, and 110 days (all before midlife) (Fig. 4H). Thus, using behavior alone, we could accurately forecast, even at a young age, whether individuals were destined to be short- or long-lived.

We next asked which specific behaviors, at a young age, might be most associated with individuals destined for a long versus short lifespan. To this end, we quantified differential behavior usage (defined as the difference between age factors in long- versus short-lived animals) over a range of ages from 50 to 150 days (Fig. 4I). Clear behavioral differences were seen between long- and short-lived animals even at young ages (Fig. 4I), but the separation became stronger as the animals aged. Focusing on 110 days (before middle age, and when behavioral classifiers are highly accurate), we observed stark differences in behaviors between long- and short-lived animals (Fig. 4, I and J). Animals destined for a long lifespan exhibited more time in vigorous or active states during the day than animals destined for a short lifespan (Fig. 4J). Additionally, individuals destined for a long lifespan, even at a relatively young age, displayed a tighter night sleep pattern, with substantially less time in sleep or rest states during the day and increased time in inactive drift and sleep states during the night than animals destined for a short lifespan (Fig. 4J). The linkage of long lifespan with well-defined circadian timing of behavior (especially sleep and inactivity-related actions) may be consistent with findings that implicate circadian timing of feeding in longevity (59, 63). Taken together, these new datasets and analyses revealed that behavior, even at a relatively young age, can forecast remaining life, with potentially major implications for identifying interventions that affect life trajectories.

## Behavioral changes with a longevity intervention: Dietary restriction

**Fig. 5.**
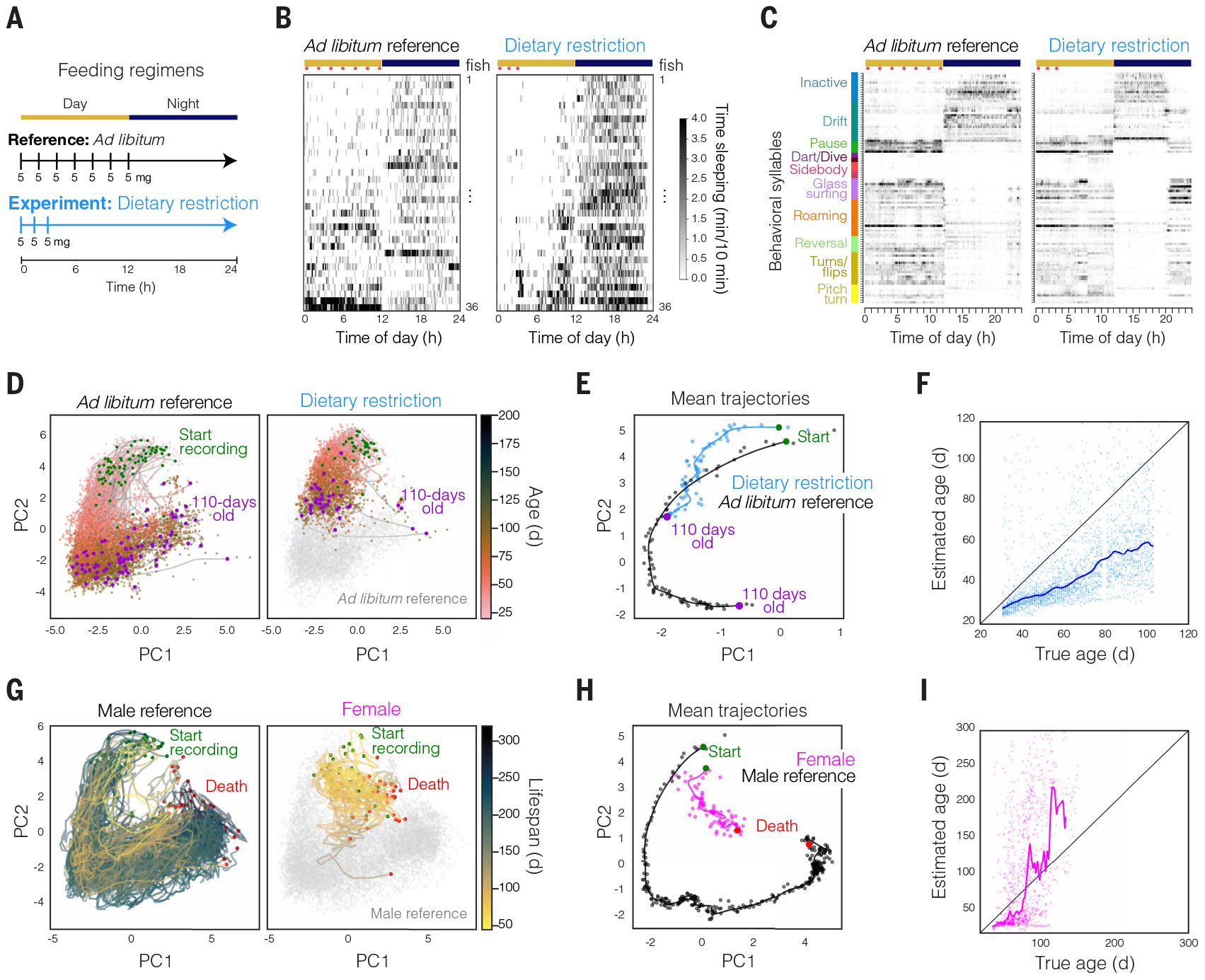
Influence of conserved longevity intervention and sex on behavior and behavioral aging trajectories. (**A**) Scheme comparing the experimental dietary restricted feeding regime with the ad libitum reference feeding regime. (**B**) Heatmap of time spent sleeping across 24 hours at 54 days old, with each row representing a different animal. Animals under dietary restriction (right; *n* = 36) are compared with randomly selected ad libitum reference animals from previous cohorts (left; *n* = 36). Red asterisks indicate feeding times, and the light-dark cycle is indicated by a yellow–dark-blue bar. (**C**) Behavioral syllable usage across 24 hours for a single animal under dietary restriction (right) and a single representative animal selected from the ad libitum reference cohorts (left) at 33 days old. See fig. S16F for mean behavioral syllable usage across all animals under each feeding regime. Behavioral syllables are ordered and clustered as in Fig. 1I. (**D**) Behavioral aging trajectories of animals under dietary restriction (right; *n* = 39) and animals of the ad libitum reference cohorts (left; *n* = 80), with each point colored by animal age. Points represent individual days, the line shows a smoothed trajectory for an individual animal, and purple points indicate when animals reached 110 days old. (**E**) Mean aging trajectory of ad libitum reference animals (black) versus dietary restriction animals (blue). (**F**) Behavioral clock model estimated age versus true age for dietary restricted animals. (**G**) Aging trajectories of females (right; *n* = 31) and male reference animals (left; *n* = 118; ad libitum feeding) colored by lifespan. Lines show smoothed trajectories for an individual animal, and red points indicate when the animals died. (**H**) Mean aging trajectory of male reference animals (black) versus female animals (magenta). (**I**) Behavioral clock model of estimated age versus true age for females.

Quantitative and noninvasive evaluation of the progression of aging in individuals provides the capability to explore how longevity interventions influence the process of aging. Longevity interventions such as dietary restriction (both calorie-restricted and time-restricted feeding) have been shown to extend lifespan and promote health in animals across the animal kingdom from *Caenorhabditis elegans* to nonhuman primates (64, 65), including in killifish (50, 66). We next asked how dietary restriction might influence behavior and progression along aging trajectories. To address this question, we tracked behavior in a cohort of male individuals (*n* = 39) undergoing a dietary restriction regime that has previously been reported to result in lifespan extension in killifish (50), with feedings only three times per day and all during the morning (Fig. 5A). Animals undergoing dietary restriction were tracked, and their behavior was analyzed from adolescence until 110 days of age. We compared the behavioral characteristics and aging trajectories of animals undergoing dietary restriction with those of prior cohorts of animals undergoing ad libitum feeding (the ad libitum reference population was fed seven times per day spaced evenly throughout the 12-hour light period; Fig. 5A).

Animals undergoing this dietary restriction regime demonstrated notable changes to the circadian timing of active and sleep behaviors (Fig. 5B and fig. S16E). Specifically, animals under dietary restriction were found to wake earlier in the morning (Fig. 5B and fig. S16E), with an increase in active behavioral syllables hours before the light change (Fig. 5C and fig. S16F). Despite their early awakening, animals undergoing dietary restriction exhibited increased total sleep duration (fig. S16C; sleep at 60 days old, *P* = 0.01, Mann-Whitney *U* test), with sleep timing more restricted to night (dark) hours (Fig. 5B). Whereas animals undergoing dietary restriction revealed no significant differences in median movement velocity (fig. S16A), these animals did exhibit significantly elevated peak velocity relative to the ad libitum reference population (fig. S16B; peak velocity at 60 days old, *P* < 0.0001, Mann-Whitney *U* test), aligned with our previous observation that lifespan is positively correlated with peak velocity at a relatively young age.

When we compared the behavioral aging trajectory of animals undergoing dietary restriction until 110 days of age with the ad libitum reference population, we observed that the two groups followed a similar aging trajectory in PC1 and PC2 but that animals undergoing dietary restriction moved along that trajectory much more slowly (Fig. 5, D and E). Indeed, when we quantified individual behavioral age using our behavioral clocks of age, we discovered that animals under dietary restriction were estimated to be behaviorally younger than their true chronological age by 42 days (median error from 100 to 110 days old) (Fig. 5F), revealing that animals undergoing dietary restriction age in a similar manner but at a reduced rate (fig. S16H; *P* < 0.0001, Mann-Whitney *U* test). This finding may be consistent with those from studies in which calorie-restricted animals (mice and nonhuman primates) die with similar pathologies and diseases as controls but at older ages (59, 67, 68). Here, we focused tracking analysis from adolescence until 110 days of age, and thus this analysis was terminated before assessment of the impact of dietary restriction on lifespan in this cohort (see Materials and methods). It will be interesting to extend this analysis to test whether the trajectory observed continues until death and whether it is linked to lifespan extension.

## Lifelong behavioral aging in females

Aging is sexually dimorphic across the animal kingdom (69), including in humans (70, 71), and it is critical to study the process of aging in both sexes. We therefore explored whether female killifish age in a similar manner to males or instead follow a distinct path through life. We tracked behavior in a cohort of female killifish (*n* = 31) from adolescence to natural death. With the feeding paradigm and husbandry conditions used for lifespan experiments, in which animals are individually housed and are not bred throughout life, we observed that killifish females are short-lived compared with males (fig. S17E). We compared the aging trajectories of females with those of our previously tracked cohorts of males undergoing matched feeding (ad libitum) and tracking conditions (“Male reference”; Fig. 5, G and H). Females progressed through a distinct aging trajectory relative to the male reference population (Fig. 5, G and H). The female behavioral aging trajectory was found to be similar to the trajectory of short-lived males (Fig. 5G) and differed markedly from the mean male trajectory (Fig. 5H). When using the behavioral aging clock (trained on male reference data), we observed that at young ages (<50 days old), females were estimated to be behaviorally younger than their true chronological age. With aging, however, females exhibited a pronounced acceleration in aging trajectory based on behavior, and females >100 days old were estimated to be 50 to 200 days older than their true chronological age (Fig. 5I). Moreover, most (74%) of the female animals were correctly predicted to be short-lived based on the behavioral classifier for lifespan at 70 days of age (fig. S17F), suggesting that some (but not all) of the behaviors that are important for longevity-model prediction are shared between females and males. Thus, our behavioral classifier for lifespan, which was trained on a male population with only 33% short-lived individuals, was nevertheless able to predict greater than twofold enrichment of short-lived females. These results suggest that this model already substantially generalizes despite the large differences between male and female aging, although this model will be extended in the future with training data from both sexes. Females exhibited lower median locomotor velocity and more time sleeping than males (but did not exhibit different peak velocity) (fig. S17, A to C); females were also smaller in size than males (fig. S17D). Overall, we discovered that killifish females progress through a distinct and accelerated aging process, consistent with our observation of shorter female lifespan in individually housed conditions (fig. S17E). These data raise the possibility that female deaths may be associated with distinct causal pathologies compared with male deaths, although further understanding of why female and male killifish age along distinct trajectories remains unresolved.

## Architecture of aging: Distinct stereotyped life stages

**Fig. 6.**
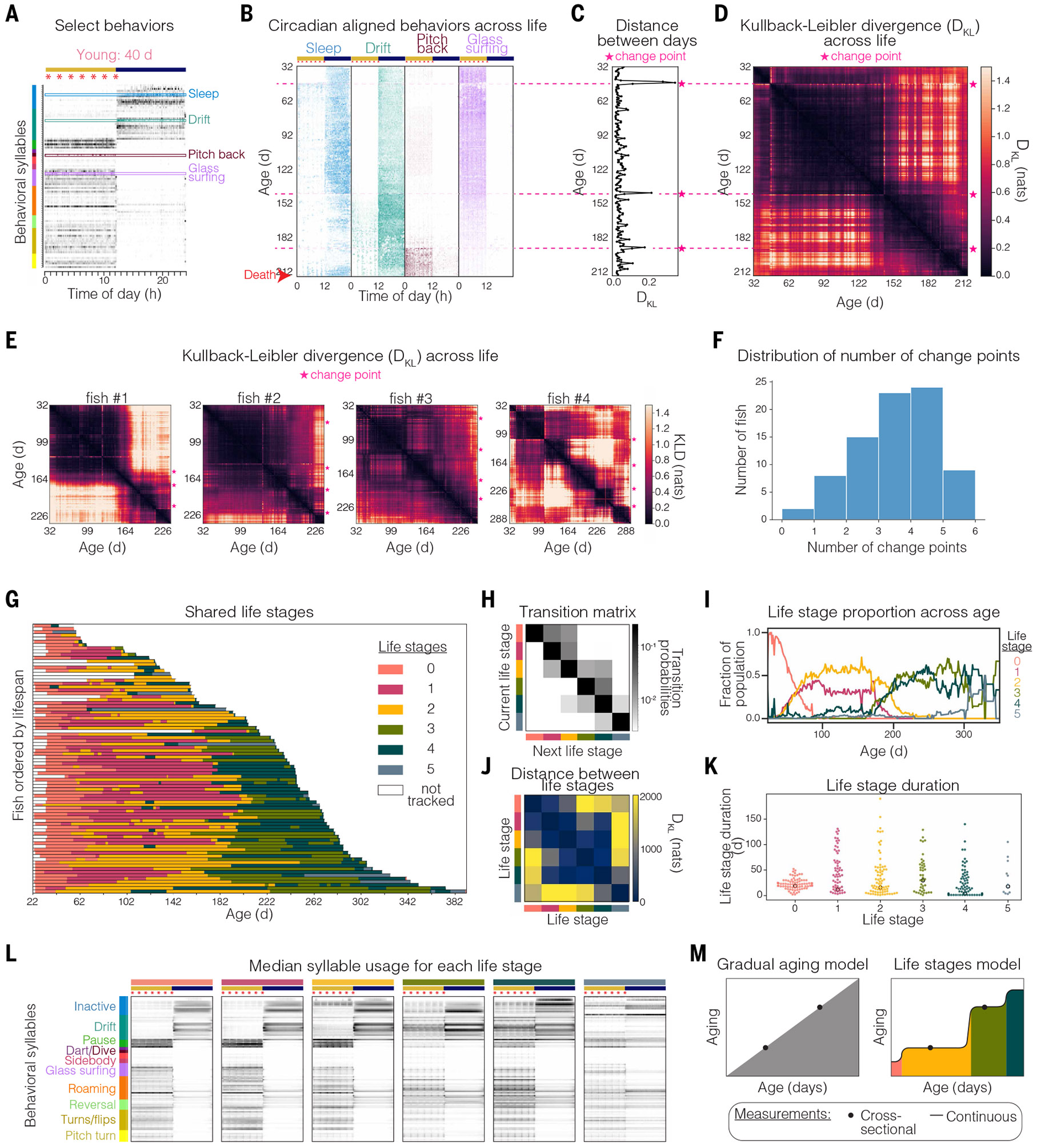
Aging animals exhibit stereotyped behavioral transitions across life. (**A**) Single-day (age 40 days) behavioral syllable use for a single animal highlighting four specific behavioral syllables. Ordering and clustering of behavioral syllables are as in Fig. 1I, red asterisks indicate feeding times, and the light-dark cycle is indicated by a yellow–dark blue-bar. (**B**) Circadian-aligned, whole-lifespan heatmaps of four select behaviors highlighted in (A). (**C**) Behavioral distance (D_KL_) of behavioral syllable usage distribution comparing subsequent days highlighting detected change points (pink star and dashed line), which aligns with specific days with abrupt transition in behavioral syllable usage. (**D**) Cross-correlation using symmetrized D_KL_ of the behavioral syllable usage distribution comparing all days in one animal’s life to all other days ordered from youth (32 days) to death (231 days). The scale of days in (C) and (D) is aligned with age in (B), and the same animal is shown in all. Change points are highlighted with a pink star. nats, natural units of information. (**E**) Cross-correlation using symmetrized D_KL_ as shown in (D) for four animals ordered by lifespan. (**F**) Distribution of the number of change points observed per animal. (**G**) Survival curve of the population of tracked animals (*n* = 81), with the coloring of each animal based on the HMM-derived life stage. (**H**) Transition probability matrix for each life stage. (**I**) Proportion of the population in each of the six life stages across age. (**J**) Symmetrized D_KL_ of HMM emission distributions across life stages. (**K**) Life-stage duration, where each point is a different animal. (**L**) Median 100 behavioral syllable usage for animals in each life stage. (**M**) Comparison of models of aging.

Continuous readout of aging from adolescence to death in a vertebrate provides an opportunity to examine and model the stereotyped progression through adult life. When each individual behavioral syllable was plotted across life with each day circadian phase–aligned, we were surprised to observe multiple discrete, sharp, and stable behavioral transitions across the lifespan (Fig. 6, A and B, and fig. S18A).

To quantify these transitions, we performed cross-correlation analysis comparing each day in an animal’s life to all other days and measured behavioral distance between days using symmetric Kullback-Leibler divergence (D_KL_) across all 100 behavioral syllables (Fig. 6, C and D, and fig. S18). We observed stark transitions at characteristic ages in each individual (Fig. 6, B to E, and fig. S18). Between abrupt transitions, we observed long periods of largely stable behavior composed of very similar days. To quantify these discrete transitions, we performed change-point detection on behavioral syllable usage across life. For the animal depicted in Fig. 6, A to D, we identified three discrete change points (highlighted in Fig. 6, C and D), which align with peaks in D_KL_ from one day to the next (Fig. 6, C and D). The first of these transitions occurred during relative youth (47 days), after which the animal shifted from exclusively sleeping during the night to exhibiting moderate daytime sleeping. The second transition occurred at more advanced age (148 days), after which the animal showed a reduction in time spent sleeping during the night. The final transition occurred close to death (192 days), after which the animal exhibited increased time spent swimming pitching backward [a likely maladaptive swimming behavior that could be due to changes in buoyancy or balance of unknown etiology (72)]. Performing the same analysis for all animals across a cohort revealed abrupt transitions in behavior across individuals, with change points occurring at slightly different ages in different animals (Fig. 6E and fig. S18D). The distribution of discrete change points (transitions) detected across all animals reveals that the vast majority of animals go through at least one transition in their adult life, and most go through three or more transitions (Fig. 6F). These observations suggested that animals progress through distinct, stable behavioral stages across the lifespan.

To investigate whether these stages are shared across animals, we modeled lifelong behavioral dynamics using a Gaussian HMM fit to the age factors. We identified six stable behavioral stages by cross-validation (Fig. 6G and fig. S19A). However, we note that the precise number of stages may depend on the model specification. These stages followed a natural order characterized by typically progressing forward, instead of switching back and forth between stages across days, as illustrated by the transition matrix of the trained HMM (Fig. 6, G and H). Given that these latent stages appear to be age associated, we adopted the term “life stages” for long-duration stages that progress forward in the orderly fashion shown. Analysis of the proportion of the population in each life stage across age illustrates that very young animals start exclusively in one young-life stage (pink), and afterward there is a split, with about half the animals entering one midlife stage (yellow) and the other half entering an alternative midlife stage (magenta). Likewise, there is a similar split into two old-life stages (dark green and light green) (Fig. 6I). Young- and midlife stages were found to be more behaviorally similar to each other than to old-life stages, as assessed by divergence (through D_KL_) between HMM emission distributions across life stages (Fig. 6J). Not all animals exhibited all six life stages, perhaps in part because life stages show variation in length across a population from a single day to >150 days (Fig. 6K).

To investigate the distinct behavioral properties of life stages, we evaluated the median daily behavioral syllable use of animals in each stage (Fig. 6L). We found that animals in young-life (pink) and midlife (yellow and magenta) stages exhibited inactive (including sleep) and drift behavior mostly at night, whereas animals in old-life stages (light and dark green) showed a shift in circadian regulation, with inactive (including sleep) and drift behavior persisting throughout both day and night (Fig. 6L). In late-life stages, there was a progressive reduction in pause behaviors. These pause behaviors typically indicate transitions from one behavior to another, suggesting that younger animals are more behaviorally flexible and dynamic (Fig. 6L). In the rare late-life stage (blue), animals exhibited a complete shift in behavior, with a few specific behavioral syllables persisting constantly throughout the day and night (Fig. 6L). Recordings of animals in this rare late-life stage (blue) reveal an age-associated swim bladder disorder that leads to disrupted buoyancy, which can be linked to infections in fish (73) (see Materials and methods). This age-associated phenotype is observed sporadically in <10% of recorded animals (and is not enriched in any cohort). Thus, our unsupervised behavioral approach can identify animals in distinct, rare states that may be linked to disease.

We also noted a correlation between an animal’s transition into late-life stages (light and dark green) and a decline in feeding behavior measured either by feeding behavior syllables or by inspection of tanks for leftover uneaten food (fig. S19B). The drop in feeding is accompanied by a range of concurrent behavioral changes—loss of circadian regulation, increased inactivity, and drift behavior during both day and night—which likely represent poor health. These periods varied in length across individuals, but most animals entered into this state substantially before death. Longer-lived animals generally spent a larger proportion of life in this state, which suggests that health span and lifespan may be uncoupled at older ages in precisely quantifiable ways with this system and that extending the healthy stages even at advanced ages (yellow and pink states in Fig. 6G) could be a well-defined and accessible quantitative target for extending human health. This life-stage model (in which aging proceeds in discrete steps or life stages, in contrast to a gradual continuous decline; Fig. 6M) would be challenging to detect with cross-sectional measurements at different ages, which supports the importance of continuous and longitudinal study of aging.

## Discussion

Here, we provide a longitudinal, continuous, high-resolution, and scalable view of the process of aging in vertebrates through behavioral screening from adolescence until death. With this dataset, we observed that animals do not all age in the same way but instead follow distinct aging trajectories. The specific trajectory an individual takes through life, as well as the speed at which that path is traversed, is influenced by sex, longevity interventions, and future lifespan. The presence of distinct types of human aging (74, 75) supports the generalizability of our observation in killifish. It may also be relevant that humans exhibit lifelong remodeling of genome structure in neurons that are crucial for defining motor and behavioral patterns (76).

We discovered that even at a relatively young age, individuals destined for a short lifespan are behaviorally distinct from individuals destined for a long lifespan. For example, at a young age, animals destined for a long lifespan exhibit higher peak locomotor velocity (“sprinting”) than animals destined for a short lifespan. Maximum velocity at middle age is highly correlated with future lifespan in *C. elegans* (77), and in humans, gait speed and decline in gait speed is associated with disability and mortality in older adults (26, 78-80). Together, these findings suggest that there are characteristics of locomotion conserved across species that could be used early in life to predict future lifespan.

We also found that by middle age, there are strong molecular signatures linked to an individual’s aging trajectory and future lifespan. Specifically, transcriptomic analysis revealed elevated liver ribosome biogenesis in short-lived individuals. This result may be particularly important because less ribosome biogenesis is linked to longevity and longevity interventions (81), whereas enhanced ribosome biogenesis and translation are observed in fibroblasts from progeria patients (82). Together, these data suggest that the amount of ribosome biogenesis even early in life could be a key regulator of lifespan.

The notable observation that short- and long-lived animals traverse divergent aging trajectories indicates that even at an early age, one can distinguish short- and long-lived individuals solely on the basis of behavior. To test this hypothesis quantitatively, we trained classification models, which we found accurately predicted an animal’s future lifespan on the basis of behavior. Behavior is highly expressive of the aging process, in line with prior work in mice indicating that behavior and frailty metrics can be used to predict age and remaining life (23, 83), but given the longitudinal nature of our dataset, we were able to show that at a young age (before midlife), behavior alone can accurately forecast an animal’s future lifespan as short versus long. In the future, it would be exciting to build models to accurately predict remaining lifespan as a continuous variable given behavior at a young age. Analogous noninvasive recording in humans [through, for example, wearable devices (84)] might provide a window into long-term health and lifespan; for example, early and accurate prediction of lifespan and age-associated disease risk could help guide early intervention (26, 84, 85).

Our view into the aging process revealed a distinctive structure in which animals stably maintain one behavioral stage for a substantial fraction of the lifespan and then transition to another distinct (and similarly stable) behavioral stage. We observed little switching back and forth between stages, noting instead an orderly progression. These shared life-stage patterns suggest an intriguing model of the aging process in which, over time, the effects of aging factors accumulate to the point that the animal must shift to a new distinct stage to reach a new stable homeostasis. This stage-based model of the architecture of aging resembles well-established models of embryonic development that include distinct staging (1-3), suggesting that perhaps such staging continues throughout life. This behavioral architecture could be driven in part by energy-conservation decisions (28), which may be best implemented in a staged manner (analogous to a gear shift in having different discrete modes rather than a continuously variable transmission).

Although humans exhibit the known discrete transitions of sexual maturity and menopause as well as sporadic health events throughout life (86), whether we can identify discrete stages of aging in human beings—analogous to those seen in killifish—remains unknown. However, supporting the notion of several life stages in humans, cross-sectional transcriptional profiling of the human frontal cortex across age revealed large changes in the fourth and eighth decades of life (87) and analysis of human plasma across lifespan revealed waves of changes in the proteome in the fourth, seventh, and eighth decades of life (88), whereas multiomic longitudinal profiling of human aging revealed nonlinear patterns in molecular markers of aging (with substantial dysregulation occurring at approximately the fourth and sixth decades of life) (89). There is also evidence of nonlinear changes to mitochondrial metabolism that may result in a staged structure of metabolic aging (90). These molecular shifts could be associated with transitions to distinct life stages [as with the genome structure changes noted above (76)], but this intriguing possibility remains to be established. Just as understanding and defining discrete staging has been fundamental to the mechanistic study of development (1-3), the staged model of aging architecture described here may provide a powerful approach to enable targeted mechanistic and therapeutic discovery work in the study of human aging.

## Material and methods

### African turquoise killifish care and husbandry

African turquoise killifish *Nothobranchius furzeri* (GRZ strain) were maintained according to established guidelines (45, 47, 91-93). Briefly, killifish embryos, originating from a breeding tank with one male (1 to 3 months) and four or five females (1 to 3 months), were raised in Ringer’s solution (Millipore, 96724), with two tablets per liter of RO water and 0.01% methylene blue (embryo solution) at 26° to 27°C in 60 mm × 15 mm petri dishes (Fisher Scientific, 07-000-328) at a density of <100 embryos per plate. After 2 weeks in embryo solution, embryos were transferred to moist autoclaved coconut fiber (Eco Earth Coconut Fiber, EE-8) lightly packed in petri dishes where they were incubated for another 2 weeks at 26° to 27°C. After 2 to 3 weeks on moist coconut fiber, embryos were hatched. For hatching, embryos were placed in humic acid solution (1 g/liter, Sigma-Aldrich, 53680 in RO water) and incubated overnight at room temperature. Once hatched, animals were housed at 26° to 27°C in a central filtration recirculating system (Aquaneering, San Diego, USA) at a conductivity between 1000 and 4000 μS/cm and a pH between 6.5 and 7.0, with a daily exchange of 10% water treated by reverse osmosis (RO water). Animals were kept on a 12-hour light/dark cycle. Young fish (pre sexual maturity) were fed freshly hatched brine shrimp (Brine Shrimp Direct, BSEP6LB) two times a day on weekdays and one time a day on weekends. Adult fish (post sexual maturity; ≥3 to 4 weeks of age) were fed dry fish food (Otohime fish diet, Reed Mariculture, Otohime C1), 5 to 7 mg per feeding, seven times a day for the ad libitum feeding paradigm or three times a day in the morning for the dietary restriction feeding paradigm using custom programable automated feeders (50). Fish were visually monitored daily for overall health. System water and tank detritus were submitted for PCR testing for aquatic pathogens. From these tests, throughout recordings, the system was negative for all *Mycobacterium* species tested with the exception of *M. chelonae* and *M. fortuitum* and also negative for *Pleistophora hyphessobryconis*, *Pseudocapillaria tomentosa*, *Pseudoloma neurophilia*, *Myxidium streisingeri*, and *Picornavirus*. During experiments including females and males under dietary restriction, bryozoans were present in the aquatic sump. They were manually removed weekly. We observed sporadic occurrence of an age-associated swim bladder disorder leading to disrupted buoyancy (commonly referred to as “belly sliders”). These animals were monitored daily to ensure they were able to swim to and consume food. There are different reports of the cause for this loss in swim bladder function. One proposed cause is due to mycobacterial infection derived from live food (for example, hatched brine shrimp) (73). Other reports find that affected animals were negative for mycobacteriosis and instead found histopathology consistent with microsporidia; subsequent sequencing analysis revealed high sequence homology with *Loma acerinae* (72). The length of lifespan of the African turquoise killifish has been reported to substantially vary between animal facilities (and within a facility over time), likely due to different husbandry conditions and other yet uncharacterized environmental parameters, which are common for a relatively recent model system (45, 47, 91-93). All animals were raised in accordance with protocols approved by the Stanford Administrative Panel on Laboratory Animal Care (protocol no. APLAC-13645).

### System for lifelong recordings

Animals were placed in standard 2.8-liter tanks (Aquaneering, San Diego, USA) in a custom-built table rack system (Fig. 1A and fig. S1). Tanks received continuous water inflow and outflow from a central filtration recirculating system (Aquaneering, San Diego, USA) with chemical and biological filtration (with water parameters described above). Each table rack was built to hold 12 2.8-liter tanks with space for opaque acrylic dividers between tanks to prevent animals in adjacent tanks from seeing one another (fig. S1). At the center of the custom rack, an acrylic gutter (Tap Plastics, San Jose, USA) was fixed for system water outflow from tanks. The rack system was built so that the top of the tanks was 2 m from the bottom of the overhead mounted camera lens (fig. S1E). The distance of the camera from the tanks is important to prevent obstruction of the tank edges. One camera was used for each 12-tank table rack for a total of three cameras and three table racks (fig. S1A).

For recordings, overhead mounted infrared-sensitive Basler industrial cameras (aca2040, monochromatic, 2048x1536, Basler AG, Germany) with attached lenses (M0824-MPW2, Computar, USA) and infrared filters (Nr.2.3) were run continuously capturing at 20 frames per second (fps) run through the Motif Video Recording System (loopbio, Austria). Custom built infrared backlighting (LLPX-750X750-850-CSM, Smart Vision Lights, USA) was fixed under the tanks (fig. S1B). Infrared backlighting and filters were used to allow for recordings throughout the 12-hour day/12-hour night lighting cycle so that circadian changes in visible light would not influence the recording itself. Tanks were covered with a custom cut red acrylic cover (Chemcast transparent acrylic one-eighth inch, Tap Plastics, San Jose, USA) to prevent animals from jumping out of tanks (fig. S1, A and B). The red color acrylic did not obstruct the infrared light on its path to the camera. The cover is largely invisible in the recordings.

Male or female killifish were individually housed in recording tanks depending on the experiment starting once they reached sexual maturity as determined by the appearance of sexually dimorphic coloration [~3 to 4 weeks of age; (94)]. Animals lived in the same tank throughout the recording period (typically until death unless otherwise noted, for example, for molecular profiling) and recordings were collected continuously (20 fps) 23 hours, 59 min, and 30 s each day. Recordings were automatically stopped at a set time each day (11:21: 43 a.m.). The recordings were then reinitiated for the next 24 hours of recording (11:22:13 a.m.). The recorded frames from the completed day were then automatically transferred from the recording computer to a storage computer. Fish were enrolled in rolling cohorts to enable the largest number of animals to be tracked and to avoid empty tanks on the recording tables resulting in 10 cohorts (cohort 1 = 24 animals; cohort 2 = 15 animals; cohort 3 = 14 animals; cohort 4 = 8 animals; cohort 5 = 8 animals; cohort 6 = 18 animals; cohort 7 = 11 animals; cohort 8 = 8 animals; cohort 9 = 10 animals; cohort 10 = 3 animals; cohort 11 = 30 animals; and cohort 12 = 39 animals). The majority of fish were recorded until natural death; however, a small number of individuals were removed prior to natural death due to (i) jumping out of their tank, (ii) removed to make space for a new cohort, or (iii) removed for cross-sectional processing such as RNA sequencing. In total, 188 fish were tracked with 121 animals tracked until natural death, 30 females and 39 males under a dietary restriction feeding paradigm.

Fish and tanks were visually monitored daily for overall health and cleanliness. If tanks showed buildup of detritus, they were manually cleaned with Plastic Pasteur Pipettes (Global Scientific, USA). Throughout recordings, key environmental parameters, for example, room lighting, room temperature, water temperature, water salinity, and water pH were monitored and regulated. Water parameters were regulated through the central filtration recirculating system (Aquaneering, San Diego, USA).

### Key-point tracking

To track animals throughout recordings we used Loopy Video Analysis and Tracking software (loopbio, Austria). Specifically, six key points (snout, midbody, endbody, tail, fan, and sidebody) along the killifish body were manually annotated in frames of video recording. Manual annotations were performed on fish of diverse age and cohort and at different times of day to ensure the training annotations would generalize well for the whole dataset. In total, 11,100 frames of individual fish were manually annotated. Not all key points were visible in each frame depending on the animal’s body positioning so the number of total annotations for each key point varied depending on frequency of key point visibility. For example, the number of annotations for each key point are as follows: 11,029 snout, 10,716 midbody, 10,609 endbody, 11,064 tail, 11,074 fan, and 466 sidebody. Manual annotations were used to train a deep convolutional neural network to predict key-point positions for fish in unseen frames using Loopy Video Analysis and Tracking software (loopbio, Austria). Trained models were used to predict key points in all frames of the recordings using Loopy Video Analysis and Tracking software (loopbio, Austria). For each frame of recording, the Loopy Video Analysis and Tracking software (loopbio, Austria) would provide the x- and y-coordinates for each visible key point as well as a corresponding timestamp for the frame. The same key-point tracking model was used for predictions across all recordings.

Each recording camera included up to 12 distinct animals because there are 12 tanks within the field of view of a single camera. All animals within the field of view were tracked simultaneously using Loopy Video Analysis and Tracking software (loopbio, Austria). Individual animal key points were separated based on the coordinates of each tank’s edge. For individual tanks, if duplicate key points were detected (for example, two snouts) due to a tracking mistake, that frame of the recording was dropped for that animal. Key-point coordinates were shifted such that the origin was the bottom left corner of each tank and key-point coordinates were rotated so that all tanks were oriented in the same direction such that the feeding side was the same for all fish.

### Pose features

Pose features were calculated from the x-/y-coordinates of tracked key points as well as the time stamp information from each frame of the recordings.

Coordinates (“x_snout,” “y_snout,” “x_midbody,” “y_midbody,” “x_endbody,” “y_endbody,” “x_tail,” “y_tail,” “x_fan,” “y_fan”). Raw x-/y-coordinates for each key point were shifted such that the origin was the bottom left corner of each tank and key point coordinates were rotated so that all tanks were oriented in the same direction such that the feeding side was the same for all fish.

Velocity (“snout_velocity,” “midbody_velocity,” “endbody_velocity,” “tail_velocity,” “fan_velocity”). Velocity was calculated for all key points after first filtering data using scipy.signal.lfilter to alleviate the impact of key point jitter.

Acceleration (“snout_acceleration”). Acceleration was calculated for the snout key point after first filtering data using scipy.signal.lfilter to alleviate the impact of key point jitter.

Dispersion (“disp”). The dispersion area is the area of the smallest bounding circle enclosing the snout key point within a defined time window. Our set time window length is 2 s (40 frames). Dispersion was calculated using the max and min x- and y-coordinate of the snout within the select time window.

Bounding area (“bounding area”). The bounding area is the smallest bounding circle enclosing all visible key points of the whole fish in a defined time window. Our set time window length is 2 s (40 frames). The bounding area is then centered at zero by subtracting the mean and normalized by the mean bounding area for that day.

Normalized body length (“body_length_norm”). Body length was calculated by summing the length of each body segment snout to midbody, midbody to endbody, endbody to tail, and tail to fan to get full body length. To calculate normalized body length, the body length was then centered at zero by subtracting the mean and normalized by the mean body length for that day.

Body segment proportions (“tail_fan_prop”). Measure of the proportion of an animal’s body length from the tail-fan segment.

Key point count (“count_snout,” “count_midbody,” “count_sidebody,” “count_endbody,” “count_tail,” and “count_fan”). Binary count of whether each key point is visible or not in a given frame (one if visible, zero if not visible). This was done for all key points.

Snout distance from tank edge (“dist_snout”). Positioning of the fish in the center versus tank edge is important in differentiating several key behaviors (for example, glass surfing behavior). Thus, we calculated the distance of the snout from the nearest tank edge.

Heading direction (“heading_direction”). The direction of the vector from midbody to snout.

Body connectivity (“alpha1,” “alpha2,” “alpha3,” “alpha_all”). The angle between body segments. For example, the angle between snout-midbody-endbody (alpha1), the angle between midbody-endbody-tail (alpha2), and the angle between the endbody-tail-fan (alpha3) body segments.

Body curvature (“theta1,” “theta2,” “theta3”). The local tangent direction at each key point – midbody (theta1), endbody (theta2), and tail (theta3) relative to heading. Tangent angles are positive when pointing toward the animal’s left side and negative when pointing toward the animal’s right side.

Reversal (“dot_product,” “reversal,” “reveral_binary”). To assess if the animal is moving forward or in reverse, we calculated the dot product of the heading direction vector and the snout velocity vector. For this, smoothing of the key-point coordinates was done first by rolling average over a 10-frame window.

Sleep (“sleep”). Sleep is behaviorally defined in zebrafish as periods of inactivity lasting 1 min or longer (95, 96). We defined inactivity based on dispersion. If the dispersion area was similar to the movement expected from key point jitter over the course of 2 s (threshold for dispersion area is 50), then the animal was determined to be inactive. If the animal maintained this inactive state for more than 1 min, it was considered sleeping.

The above features were calculated across all frames. Then, depending on the feature, the mean and/or standard deviation of each was evaluated over a rolling window of 10 frames (data S1) resulting in 57-dimensional vector per frame describing all pose features. To quantify whether distinct pose features were used during day/night or across different ages (Fig. 1G) for select animals, we measured linear separability as classification accuracy of a linear kernel support vector classifier fitted on the top five pose feature PCs using sklearn.svm.SVC with kernel=“linear” (97). For pose feature PCA, we standardized (z-scored; subtracted mean and divided by standard deviation) the pose features prior to PCA (sklearn.decomposition.IncrementalPCA).

### HMM for behavioral syllables

We infer stereotyped, reused behavioral syllables from pose features by fitting an HMM to a dimensionality-reduced representation of the pose features. The unprecedented amount of data (>30 billion frames) made it prohibitive from both a memory and computational time perspective to apply standard algorithms for fitting to the full dataset. The data, however, are highly autocorrelated across days and for a given individual. This supports the more data efficient approach of building training and validation sets on representative subsets of the data. The training set was built from a random selection of 200 full days of recording from different individuals, different cohorts, and at different ages (3.46 × 10^8^ frames at 20 fps resolution). This training set size still required incremental, distributed, and stochastic variations of algorithms, as described below. The validation set consists of recordings drawn from across ages but from individuals not present in the training set (2.1 × 10^7^ frames at 20 fps resolution).

Given the redundancy of many of the pose features and to improve the efficiency of HMM training, we first standardized (z-scored; subtracted mean and divided by standard deviation) the pose features and then performed PCA (sklearn.decomposition.IncrementalPCA). The top 15 PCs were found to explain >80% variance (fig. S2A) and the standardized pose features were reduced down to these PCs. The resulting features were smoothed with a 10-frame moving average filter.

Finally, a Gaussian HMM was fit to these preprocessed PCs to infer the underlying discrete hidden states, which are interpreted as “behavioral syllables.” The Baum-Welch algorithm (98) is the standard expectation-maximization (EM) algorithm for estimating the maximum likelihood of parameters in HMMs, but each iteration requires memory that is linear in the number of frames and quadratic in the number of states, that is, *O*(*TK* + *K*^2^), for *T* frames and *K* hidden states (>138 GB per state for each training iteration for 200 full days of recording). Note that in regimes like ours, in which *T ≫ K*, this simplifies to *O*(*TK*). To reduce the memory footprint of the standard implementation, which is prohibitive even for the reduced size of the training dataset, we developed a custom JAX implementation of stochastic EM for HMMs (99, 100). This reduces the memory footprint to *O*(*BK*^2^), for batch size of *B* sequential frames, where *B ≪ T* and is adjustable based on machine constraints. This implementation also supports parallelization across batches, reducing the time complexity linearly in the number of parallel jobs. At each iteration, we run the forward-backward algorithm to infer the hidden states for a batch of size *B* using the current model parameters. Then, we perform a Robbins-Munro style update of the parameters by taking a convex combination of the previous parameters and the optimal parameters for the hidden states of the current batch. Our implementation using 71 batches required 2 days for computation using 16 GB/CPU and 8 CPU on a single node to fit an HMM with 100 hidden states. The custom implementation is available at https://github.com/lindermanlab/scalable-gaussian-hmm.

The primary hyperparameter in this model is the number of HMM states. We selected this hyperparameter by cross-validating against the log likelihood of the held-out validation dataset under the fitted model. Held-out log likelihood was observed to begin plateauing at 100 states (fig. S4A); model training was prohibitively slow for greater than 100 hidden states, so 100 hidden states were used for analysis. Behavioral syllables were predicted for the whole dataset using the fitted HMM for downstream analysis. The distribution of syllable duration varied depending on the syllable. We evaluated mean, 95th percentile, and 5th percentile syllable duration for all 100 syllables on single days comparing young (45 days) versus old (270 days) (fig. S4G) based on nine animals (five young and four old) presented in Fig. 1G.

### UMAP embeddings to visualize pose features

UMAP embeddings of the top 15 PCs describing killifish pose features were used for visualization. Embeddings were built for select animals on a select day to build visualizations in Fig. 1, G and H, and figs. S3 and S4, C to E. Due to the large number of frames, embeddings were built using one in every five frames (down-sampling from 20 to 4 Hz frame rate) of the select data to avoid memory limitations. Embeddings were built using the top 15 PCs as described above (not smoothed) with the umap python package with n_neighbors=15, min_dist=0.3, n_components=2, random_state=42, metric=“euclidean.”

### D_KL_ analysis

D_KL_ analysis (relative entropy) was performed to calculate the pairwise dissimilarity. We use D_KL_ in three applications in the paper: (i) measuring dissimilarity of HMM-defined behavioral syllable emission distributions to establish behavioral syllable clusters (fig. S4B), (ii) measuring dissimilarity of behavioral syllable usage distributions from each day in an animal’s life compared to all other days of the same animal’s life (Fig. 6, C to E), and (iii) measuring dissimilarity of HMM-defined life stage emission distributions (Fig. 6J).

For (i), first HMM-defined behavioral syllable emission distributions were built from the behavioral syllable emission mean and covariance using torch.distributions.MultivariateNormal. The pairwise KL divergence across all 100 behavioral syllables was calculated using torch.distributions.kl.kl_divergence. We then calculated symmetrized *D*_*KL*_ = _2_^1^ *KL*( *p*∣*q*) + _2_^1^ *KL*( *q*∣*p*).

For (ii), to calculate the daily behavioral syllable usage distribution for a given day and for a given animal, we summed the total time spent in each of the 100 behavior syllables during the 12-hour light period and the 12-hour dark period to generate a 200-element long vector. This vector describes the daily behavioral syllable usage distribution for a given day in each animal’s life. D_KL_ between each day in an animal’s life was then calculated for all possible pairwise combinations of days within a single animal using the scipy.stats.entropy module. We then calculated symmetrized D_KL_ as described above.

For (iii), HMM-defined life stage emission distributions were built from the life stage emission mean and covariance using torch.distributions.MultivariateNormal. The pairwise KL divergence across all six life stages was calculated using torch.distributions.kl.kl_divergence. We then calculated symmetrized D_KL_ as described above.

### Behavioral syllable clustering

To aid in interpretation and visualization of behavioral syllables, we perform hierarchical clustering of the behavioral syllable emission distributions. Hierarchical clustering was performed using sklearn.cluster.AgglomerativeClustering with distance calculated using symmetrized D_KL_ between syllable emission distributions (described above). The resulting clusters are visualized in fig. S4B.

### TCA

For dimensionality reduction of daily behavioral data, we used TCA, which is closely related to canonical polyadic (CP) decomposition (101), following methods and software described in detail in Williams *et al*. (55) (https://github.com/neurostatslab/tensortools/). We first binned the data at 10 min resolution by counting the occurrence of each syllable over 12,000 frames (10 min). We chose to use 10 min binning because at this resolution we were able to capture key circadian events such as each feeding and the abrupt response to the light change while still reducing the size of the data for downstream processing. Non-negative TCA models were trained using the 100 behavioral syllables binned at 10 min resolution for all animals and all days of recording. Usage traces for each behavioral syllable were normalized to range between zero and one across the whole dataset to avoid bias due to highly used behavioral syllables. Data were organized into an *N* × *T* × *K* tensor with *N* = 100 behavioral syllables recorded at *T* = 144 time bins/day over *K* = number of animals × number of days of recording/animal. We then fit non-negative tensor decomposition models using normalized behavioral data. To select the optimal number of components, we performed cross-validation by holding out 50% of time-bins from the dataset and varying the number of tensor components from 2 to 100. As described by Williams *et al.*, we normalize the reconstruction error between zero and one to provide a metric analogous to the fraction of unexplained variance often used in PCA (Fig. 2B) (55). To evaluate models, the normalized reconstruction error was calculated for both the training and the held-out set. We selected a 45-component model for downstream analysis.

TCA approximates the data as a sum of outer products of three vectors—in our case, (a) time-of-day factors, (b) behavioral-syllable factors, and (c) age factors, as shown in Fig. 2, A and D. Time-of-day and behavioral-syllable factors constitute structure that is common across all days in the dataset. What makes one day different from another for each animal is the amplitude or weighting of each time-of-day and behavioral-syllable factor, that is, the age factor. The age factor can be thought of as the amplitude/weight for a given animal at a given age in the daily behavior pattern. Some TCs ramp up or down during the day and night (for example, TC19, TC22, TC41), whereas other TCs show constant weighting throughout the day and night, with no circadian regulation (for example, TC24) (subpanel a in Fig. 2D). Behavioral syllable factors vary from sparse (few syllables dominating, for example, TC34) to broad (with many behavioral syllables making small contributions to build the component, for example, TC43) (subpanel b in Fig. 2D). One tensor component (TC20) peaks at each of the seven feedings and is composed of behavioral syllables that represent feeding behavior (although additional direct measures of food consumption would be necessary to explore feeding in a more finely resolved manner). Two TCA models were trained, one model including tracking data from all males under the ad libitum feeding paradigm and a second model additionally including tracking data from females and males under dietary restriction. Age factors for the first model (including only males under the ad libitum feeding) are presented in Figs. 2 to 4 and 6, whereas the second model age factor weightings for all animals are presented in Fig. 5.

### Aging trajectories

To visualize aging trajectories (that is, the change in age factor weighting across life) for each animal, we performed PCA of TCA-derived age factors. Prior to PCA, we z-scored the TCA-derived age factors. We visualized the top three PCs (~40% explained variance; fig. S7A) in Figs. 2, E to G; 3, B and H; and 5, D, E, G, and H; and figs. S7B; S8; S9; S11, B and C; and S16G. For visualization smoothed trajectories, we used scipy.ndimage.gaussian_filter. For visualization purposes only, we also performed UMAP embeddings of TCA-derived age factors. To build UMAP embeddings, we used the top 20 PCs (described above) using the umap python package with n_neighbors=15, min_dist=0.3, n_components=2, random_state=42, metric=“euclidean.” The resulting visualizations are shown in fig. S10. To evaluate how similar behavioral aging trajectories are across individuals, we quantified the summed variance of all 45 TCs at each age across life (fig. S10A).

### Organ harvesting

Animals were fasted for 24 hours before organs were harvested. RNA sequencing was performed on eight organs (brain, heart, intestine, kidney, liver, skin, spleen, and testis) in wild-type animals at different ages: young (80 days; *n* = 8), middle-aged (150 days; *n* = 17), and old (210 days, *n* = 4). Each cohort was tracked starting at adolescence until the designated endpoint. Organs were harvested in the morning. Organs were dissected on ice-cold Sylgard-coated Petri dishes (filled with ice and covered in plastic wrap) and were snap-frozen in liquid nitrogen after harvesting (stored at −80°C until RNA isolation). Skin samples were collected from the caudal fin.

### RNA isolation

RNA was isolated from organs using QIAGEN RNeasy Mini kit (QIAGEN, 74106), following protocols previously described (50). Briefly, organs were transferred to 1.2 ml Collection Microtubes (QIAGEN, 19560), on dry ice to reduce tissue thawing. Autoclaved metal beads (QIAGEN, 69997) and 700 μl of QIAzol (QIAGEN, 79306) were added to each tube followed by tissue homogenization on a TissueLyserII machine (QIAGEN, 85300) at 25 Hz, 5 min each, at room temperature. Lysates were transferred to 1.5 ml tubes and 140 μl chloroform (Fisher Scientific, C298-500) was added followed by vortexing and incubation at room temperature for 2 to 3 min. Lysates were then centrifuged at 12,000*g* at 4°C for 15 min. The aqueous phase was mixed with 350 μl ethanol (200 Proof, Gold Shield Distributors, 412804) and then transferred to RNeasy columns from the RNeasy RNA Purification Kit (QIAGEN, 74106). The RNeasy column was then washed with 350 μl RW1 buffer (provided by the RNeasy Mini kit), and treated with DNase I (following the kit’s protocol) at room temperature for 15 min. The column was washed two times with 500 μl RPE buffers, and the RNA was eluted with 50 μl nuclease-free water (Invitrogen, 10977023). RNA quality and concentration were measured using an Agilent 2100 Bio-analyzer and the Agilent Nano Eukaryotic RNA Kit (Agilent, 5067-1511). All bioanalyzer assays were performed by the Stanford Protein and Nucleic Acid Facility.

### Multiorgan RNA sequencing analysis

#### cDNA library generation and sequencing using BRB-seq for RNA barcoding:

Organ RNA samples were prepared following manufacture recommendations for the BRB-seq platform with the Mercurius Protocol (Alithea Genomics) for bulk RNA barcoding and sequencing. Briefly, RNA concentration was determined by using the Qubit 1X dsDNA High Sensitivity Assay Kit (Thermo Fisher, 797 Q33231) and between 0.48 and 0.66 μg of total RNA from each sample were reverse transcribed in a 384-well plate with unique barcoded oligo-dT primers. Amount of loaded RNA was matched between all samples of a given tissue but the amount varied between different tissues based on yield from RNA isolation (brain-0.66 μg; gut-0.66 μg; heart-0.48 μg; kidney-0.66 μg; liver-0.66 μg; skin-0.48 μg; spleen-0.48 μg; testis-0.66 μg per sample). For sample pooling, 10 μl of each barcoded reverse transcribed sample were pooled and purified using Zymo Clean and Concentration Kit (Zymo, D4014) column and DNA Binding buffer (Zymo, D4004-1-L) followed by free primer digestion. Double-stranded cDNA was generated by second-strand synthesis via the nick translation method. The concentration was measured by Qubit (190 ng/μl), and prepared for tagmentation using the Tn5 transposase pre-loaded with adapters for library amplification. The cDNA library was prepared for Illumina sequencing and sequenced on an Illumina NovaSeq 6000 (2 × 150 bp paired-end) by Novogene (Novogene, Beijing, China), at a sequencing depth of >16 million pair-end reads per sample.

#### RNA-seq data processing pipeline:

Adaptors were first trimmed from raw sequencing FastQ files using Cutadapt (version 3.1) for removing the last 122 bases of each Read 1 sequence (with parameters -u -122) followed by quality assessment using FastQC. Processed reads were then aligned to the African turquoise killifish reference genome [Nfu_20140520, GCF_001465895.1 (39)] and the gene read count and UMI count matrices were created using STAR (version 2.7.1a) with parameters adjusted according to recommendations for the BRB-seq platform with the Mercurius Protocol (Alithea Genomics) including:

–*soloCBwhitelist*: text file with list of barcodes used by STAR for demultiplexing (see data S2).

–*soloCBstart*: start position of the barcode in the R1 fastq file = 1.

–*soloCBlen*: length of barcode = 14

–*soloUMIstart*: start position of UMI = 15

–*soloUMIlen*: length of UMI = 14

The sequenced library had 86.3% of reads mapped uniquely to the genome. Raw gene expression values were then normalized using DEseq2 (version 1.36.0) excluding genes with <20 counts across all samples. For differential expression analysis between young (80 days) and old (≥210 days) animals, we used DEseq2 with the design “~ age” and then evaluated the differences between young (80 days) and old (≥210 days) animals. For differential expression analysis between predicted short-lived and long-lived behavioral trajectories, we used DEseq2 with the design “~ trajectory” for only the middle aged (150 days) data and then evaluated the differences between predicted short- versus long-lived behavioral trajectory animals (data S2). PCA was performed after filtering out genes with <20 counts across all samples followed by variance stabilizing transformation (vst function in the DEseq2 package) (Fig. 3I and fig. S13A). The resulting filtering led to the following gene numbers per tissue: 18,723 (liver), 20,773 (brain), 19,335 (intestine), 19,158 (heart), 19,995 (kidney), 18,725 (skin), 20,070 (spleen), and 21,789 (testis).

#### Enrichment analysis:

To perform overrepresentation analysis for significantly differentially expressed genes between either young (80 days) versus old (≥210 days) or short-lived versus long-lived trajectory (all 150 days old), we used GO enrichment analysis using the enrichGO function in the clusterProfiler package (version 4.4.4) (data S2). For young versus old, we ran analysis on genes significantly up-regulated (padj <0.05 and log2FoldChange >0) and significantly down regulated (padj <0.05 and log2FoldChange <0). For short-lived versus long-lived trajectory, we ran analysis on genes significantly up-regulated (pvalue <0.05 and log2FoldChange >0) and significantly down regulated (pvalue <0.05 and log2FoldChange <0). For gene set enrichment analysis, we used GO analysis using the gseGO function in the clusterProfiler package (version 4.4.4) (data S2). Genes were ranked by sign(log2FoldChange)*[−log10(padj)]. Each killifish gene was then assigned to its human ortholog (best hit protein with BLASTp E-value >1 × 10^−3^). Killifish genes with no human ortholog were removed. Where multiple killifish paralogs were present for a single human ortholog, the killifish paralog with the smallest adjusted *P* value was kept. Enrichment analysis was run using the Bioconductor annotation data package (org.Hs.eg.db v3.15.0). The *P* values of the enriched pathways were corrected for multiple hypotheses testing using the Benjamini-Hochberg method.

#### Clustering and separability of transcriptomes of short- versus long-lifespan trajectory animals:

To evaluate separation of transcriptomes of the short- versus long-lifespan trajectory animals across all tissues, we measured silhouette score (sklearn.metrics.silhouette_score) in PC1/PC2 space of true labels (long-lifespan versus short-lifespan trajectory based on behavior) compared to a null distribution built by permuting randomized group assignment across all *n* = 17 samples to two groups and evaluating the resulting silhouette score (fig. S13B). We also performed unbiased k-means clustering of the liver PC1/PC2 data using sklearn.cluster.KMeans with the number of clusters set to two and default parameters which resulted in two clusters that matched the observed short-lifespan and long-lifespan trajectory labels (fig. S13C).

### Regression models to estimate age

Our goal was to build models to accurately estimate animal age given the behavioral characteristics of the animal on a given day. Models were designed so that each day is described by a vector of the 45 TCA-derived age factors used as input features (no smoothing or standardization was performed) and then predict the animal’s age (that is, estimate age). We performed leave-one-fish-out cross-validation where one fish in the training set was held out, the model was trained on all days for remaining fish using input features and age label, and then age estimates were made on the held-out fish based on input features (Fig. 4, B and C). For cross-validation, random forest regressors were built using sklearn.ensemble.RandomForestRegressor with default parameters. To evaluate model performance, we evaluated correlation of model estimated age with true age for all ages of held-out fish using Pearson correlation with numpy.corrcoef (Fig. 4B). We evaluated the absolute value of the error of held-out animal age estimates versus true age for each age tested and then calculated the median absolute error across all animals for each age (Fig. 4C).

We also trained random forest regressors and evaluated model performance on a held-out test set of animals. For model training, random forest regressors were built using sklearn.ensemble.RandomForestRegressor with n_estimators = 500 and default parameters. Held-out test-set animals came from three separate cohorts run at different times on the tracking system including cohort 5 (eight animals tracked for their whole lifespans), cohort 6 (18 animals tracked until 4 months old), and cohort 9 (10 animals tracked for their whole lifespans) for a total of 36 held-out animals. No animals from any of these cohorts were used for model training. Model performance was then evaluated by correlation (fig. S15B) and median absolute error (fig. S15C) as described above. To investigate which of the 45 TCA-derived age factors were most important for model predictions, we measured the mean decrease in impurity using the sklearn.ensemble. RandomForestRegressor attribute feature_importances_ on the trained regressor (Fig. 4D).

To estimate age in cohorts of males under dietary restriction and females, because these datasets were analyzed using a distinct TCA model, we trained new random forest regressors trained as described above on data from the second TCA model (using sklearn.ensemble.RandomForestRegressor with n_estimators = 500 and default parameters). For training, we held-out a test set of male animals under ad libitum feeding described above (cohort 5, *n* = 8 animals tracked for their whole lifespans; cohort 6, *n* = 18 animals tracked until 4 months old; cohort 9, *n* = 10 animals tracked for their whole lifespans) as well as all males exposed to dietary restriction (*n* = 39 animals tracked until 4 months old) and all females (*n* = 30 animals tracked for their whole lifespan) such that the model was trained exclusively with data from males under ad libitum feeding. Model performance was then evaluated on the held-out test set of males under ad libitum feeding by both Pearson correlation (*R* = 0.93) and median absolute error across all ages (14 days). This model was then used to estimate age of males under dietary restriction (Fig. 5F) and females under ad libitum feeding (Fig. 5I).

To compare the rate of aging of individual animals we calculated the best fit linear slope of behavior clock estimated age versus true age (using scipy.stats.linregress) for each individual across all days recorded. We then compare the DR fed animals to the ad libitum fed animals. For the ad libitum fed animals we used a held-out test set of animals not used for behavior clock training. DR fed animals had a significantly slower rate of aging compared to the ad libitum fed animals (*P* < 0.0001 Mann-Whitney test; fig. S16H).

### Classification model to forecast future lifespan

Our goal was to build models to accurately forecast an animal’s future lifespan as either long-lived or short-lived based on their behavior. To do this, we built separate classification models at ages throughout the killifish lifespan. We chose to build age-fixed classification models to detangle the impact of age on prediction accuracy given that age alone is a relatively good predictor of remaining life. To avoid day-to-day noise in behavior, we built each age-specific model taking the mean behavior over a set window of days (a 5-day window). Thus, each age-specific model was designed to use the mean of the 45 TCA-derived age factors over a 5-day window prior to the prediction age as input features and then classify the animal as either short-lived or long-lived. Input features were standardized (z-scored; subtracted training set mean and divided by training set standard deviation). We defined animals as being long-lived if they lived equal to or longer than 200 days (33rd percentile). We selected 200 days as the cutoff as this represented a significant deviation from the median lifespan of the population (243 days) but a large enough fraction of the population for model training. We excluded extreme agers—animals that lived for >300 days (90th percentile). For age-specific classifiers, we used random forest classifiers with sklearn.ensemble.RandomForestClassifier with default parameters. Each age-specific model was trained using leave-one-fish-out cross-validation (as described above). Leave-one-fish-out model performance was evaluated by prediction accuracy (using sklearn.metrics.accuracy_score) and area under the receiver operating characteristic curve (ROC AUC using sklearn.metrics.roc_auc_score) for each age-specific model (Fig. 4, F and G). To evaluate the level of separation between the model predicted short-lived versus long-lived groups, we grouped animals by held-out predictions at a set prediction age and then plotted the measured Kaplan-Meier survival curves of these two populations of animals using lifelines.KaplanMeierFitter (Fig. 4H). This was done for three select prediction ages: 70, 90, and 110 days. We then evaluated whether the predicted short-lived and predicted long-lived groups have a significant separation in true lifespan using a log-rank test (lifelines.statistics.logrank_test).

To better understand the behavioral differences in animals destined for a short lifespan versus long lifespan, we performed differential behavioral analysis between short-lived and long-lived animals across ages from 50 to 150 days old. For this analysis, we measured the mean behavior of short-lived animals and long-lived animals (that is, the 45 TCA-derived age factors) across age. Significance was determined using Mann-Whitney *U* test with Bonferroni correction (with scipy.stats.mannwhitneyu and statsmodels.stats.multitest.multipletests) (Fig. 4I).

To classify lifespan in females, because these datasets were analyzed using a distinct TCA model, we trained a new age-specific (70 days old) random forest classifier trained as described above on data from the second TCA model using sklearn.ensemble.RandomForestClassifier with default parameters. Model performance was evaluated by leave-one-fish-out predictions based exclusively on behavior of males under ad libitum feeding by both prediction accuracy = 0.77 (using sklearn.metrics.accuracy_score) and ROC AUC = 0.72 (using sklearn.metrics.roc_auc_score). This model was then used to classify lifespan of females based on behavior at 70 days old (fig. S17F).

### Change-point detection

To evaluate the number of discrete transitions in behavior across life, we used change-point detection analysis to identify discrete times within individual aging trajectories in which there was a shift in underlying characteristics of the time series. We performed change-point detection on daily behavioral syllable usage across life. To calculate the daily behavioral syllable usage distribution for a given day and for a given animal, we summed the total time spent in each of the 100 behavior syllables during the 12-hour light period and the 12-hour dark period to generate a 200-element long vector. This vector describes the daily behavioral syllable usage distribution for a given day in each animal’s life. We smoothed daily behavioral syllable usage to alleviate the impact of daily noise using scipy.ndimage.gaussian_filter with sigma = 1 and then standardized the smoothed age factors (z-scored; subtracted mean and divided by standard deviation). We performed change point analysis using the python package rupture; https://github.com/deepcharles/ruptures (102). Kernel change-point detection (103, 104) was performed with a Gaussian kernel (using rupture. KernelCPD), an unknown number of change points per animal, and a penalty that identically scales with the natural log of lifespan length across all animals. We indicated change points in individual example animals in Fig. 6, C to E. We evaluated the number of discrete change points observed across the life of each animal, and the plot of the distribution of change point number per animal across the whole population is shown in Fig. 6F.

### HMM for life stages

To build the life-stage model, we first smoothed the TCA-derived age factors to alleviate the impact of daily noise using scipy.ndimage.gaussian_filter with sigma = 1 and then standardized the smoothed age factors (z-scored; subtracted mean and divided by standard deviation). We trained Gaussian HMMs with *k*-means initialization and iterated until convergence (*n* = 20 iterations of expectation-maximization) using Dynamax (a library for probabilistic state space models written in JAX: https://github.com/probml/dynamax). We varied the number of HMM states, and we observed that the held-out log probability plateaued around five states and dropped off sharply at seven states (fig. S19A). The resulting life-stage model with six states accurately discriminated a rare life stage when animals are affected by a swim bladder disorder leading to disrupted buoyancy which was missed in the five-state model. Given the biological relevance of this rare sixth state, we chose to move forward with a six-state HMM for life-stage modeling. Using full covariance versus diagonal covariance Gaussian HMMs could yield a different number of stages.

We next investigated the characteristics of this six-state life-stage model. To evaluate the empirical transition matrix of the trained HMM, we counted the frequency of each state-to-state transition across all animals in the cohort (Fig. 6H). To evaluate the length of each life stage, we calculated the empirical duration of each bout in each life stage across all animals and evaluated median duration in each life stage (Fig. 6K). To investigate the behavioral syllable signatures of each life stage, we grouped days by life stage and evaluated median time in each behavioral syllable across all time bins throughout the 24-hour day for each life stage. This was done with 10-min binned behavioral syllable data (144-time bins per day) (Fig. 6L).

## Supplementary Material

Bedbrook Supplement

Movie_S1

Movie_S2

Data_S1

Data_S2

MDAR_Reproducibility_Checklist

science.org/doi/10.1126/science.aea9795

Figs. S1 to S19; MDAR Reproducibility Checklist; Movies S1 and S2; Data S1 and S2
