# Supplementary material for: Lifelong behavioral screen reveals an architecture of vertebrate aging
Source: Science. Author manuscript; Available in PMC 2026 May 12. (PMC13165398; doi:10.1126/science.aea9795)
Supplement: Bedbrook Supplement [file NIHMS2164217-supplement-Bedbrook_Supplement.pdf]

Science AAAS

Supplementary Materials for

Lifelong behavioral screen reveals an architecture of vertebrate aging

Claire N. Bedbrook *et al*.

Corresponding authors: Anne Brunet, abrunet1@stanford.edu; Karl Deisseroth, deissero@stanford.edu

Science **391**, eaea9795 (2026)
DOI: 10.1126/science.aea9795

The PDF file includes:

Figs. S1 to S19

Other Supplementary Material for this manuscript includes the following:

MDAR Reproducibility Checklist
Movies S1 and S2
Data S1 and S2

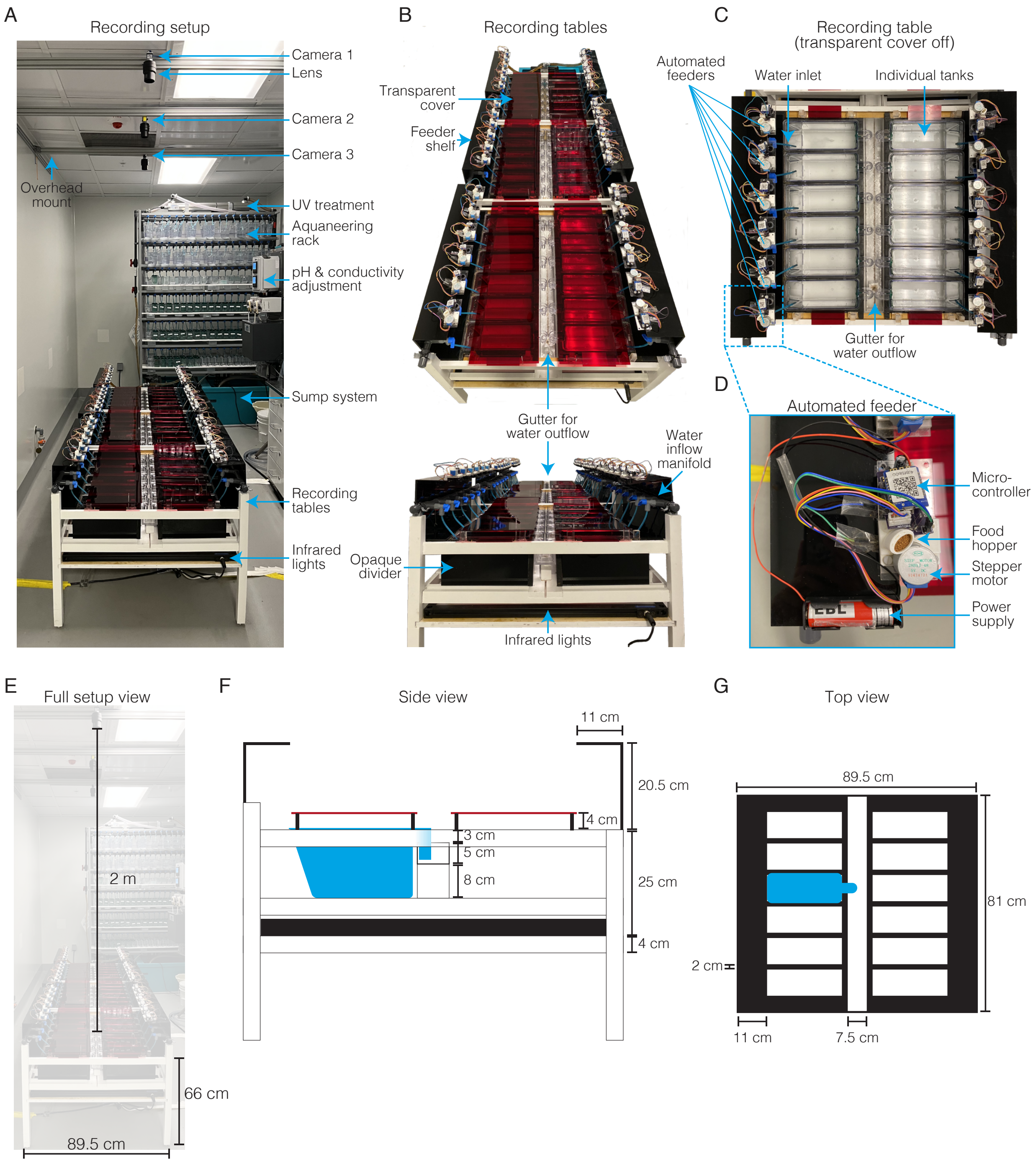

Fig. S1. Long-term recording setup. (**A**) Three custom table racks with continuous water inflow and outflow from Aquaneering rack and sump system. Three overhead mounted infrared-sensitive cameras (one per table). (**B**) Twelve 2.8-L tanks fixed in each custom table rack. Infrared-transparent red acrylic lid covers tanks. Automated feeders mounted on a black acrylic shelf drops food into tanks at fixed times each day. Gutter collects tank outflow for filtering and recirculation through sump system. Water inflow manifold fixed to table racks with inlet valves and inlet tubes for each tank. Opaque black acrylic dividers between tanks prevents fish in adjacent tanks from seeing one another. Infrared backlight below tanks. (**C**) Overhead view of

single table rack without acrylic cover. (**D**) Zoom in on automated feeder highlighted in (**C**) showing feeder components. (**E**-**G**) Measurements of the setup.

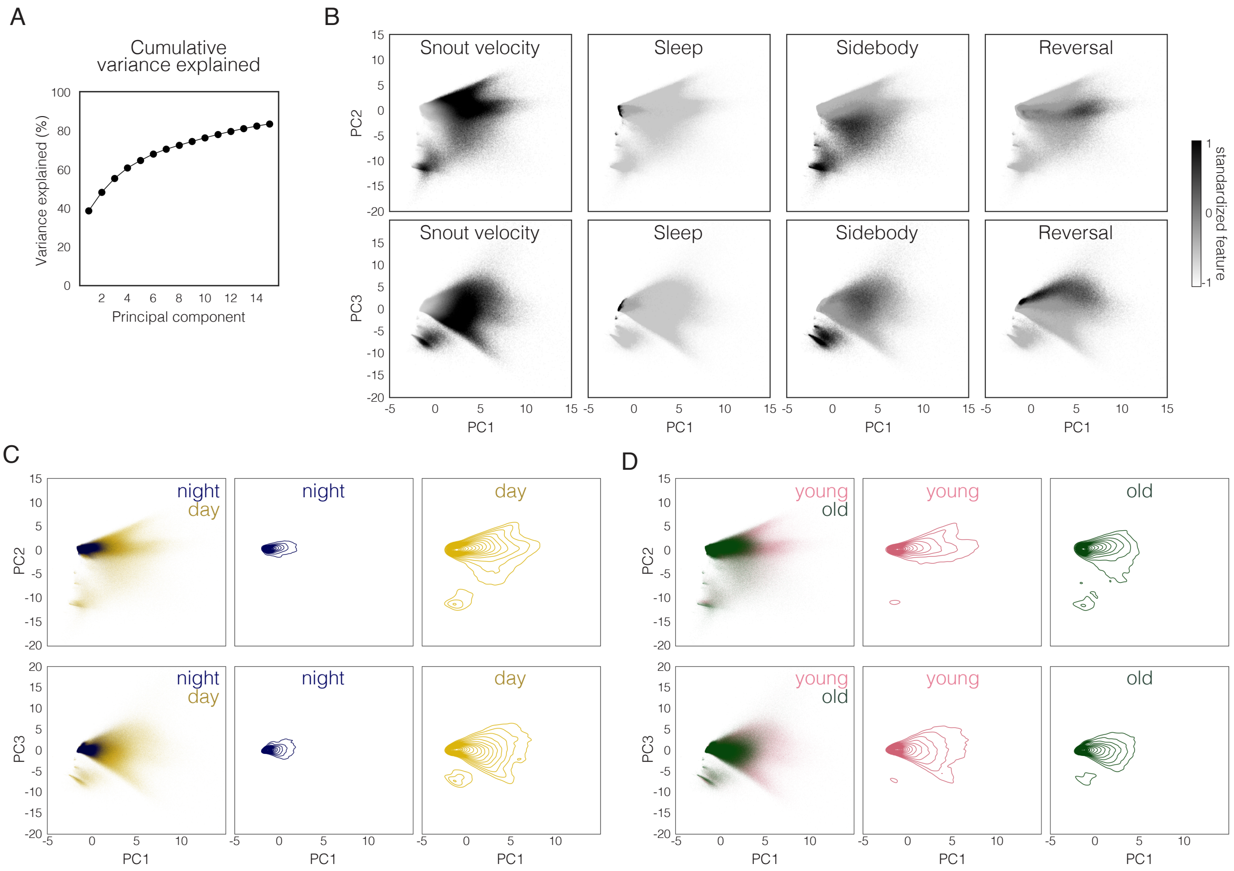

Fig. S2. PCA of pose features. (**A**) Cumulative variance explained by up to 15 principal components used for training HMMs. (**B**) Top three pose feature PCs (PC1, PC2, PC3) of data from a single day (down-sampled from 20 Hz to 4 Hz frame rate) for nine different fish at two ages (45 days old and 270 days old). Each scatter point represents a single frame of recording and color maps indicating heatmap for four select pose features. (**C**) Same as (**B**) but colored by day (yellow) vs. night (blue) frames (left) and contour plots of frames separating day and night (right). (**D**) Same as (**B**) but colored by colored by young (pink: 45 days old) vs. old (green: 270 days old) frames (left) and contour plots of frames separating young and old (right).

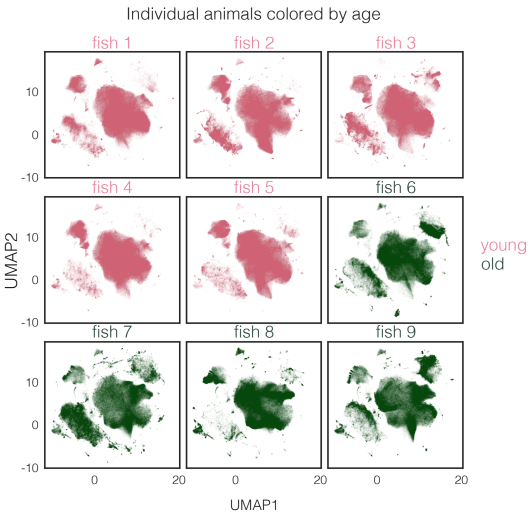

Fig. S3. Individual animal pose features. UMAP embedding of pose features PCs of data from a single day (down-sampled from 20 Hz to 4 Hz frame rate) for nine different fish at two ages (pink: 45 days old, young; and green: 270 days old, old). Each scatter point represents the embedding of a single frame of recording.

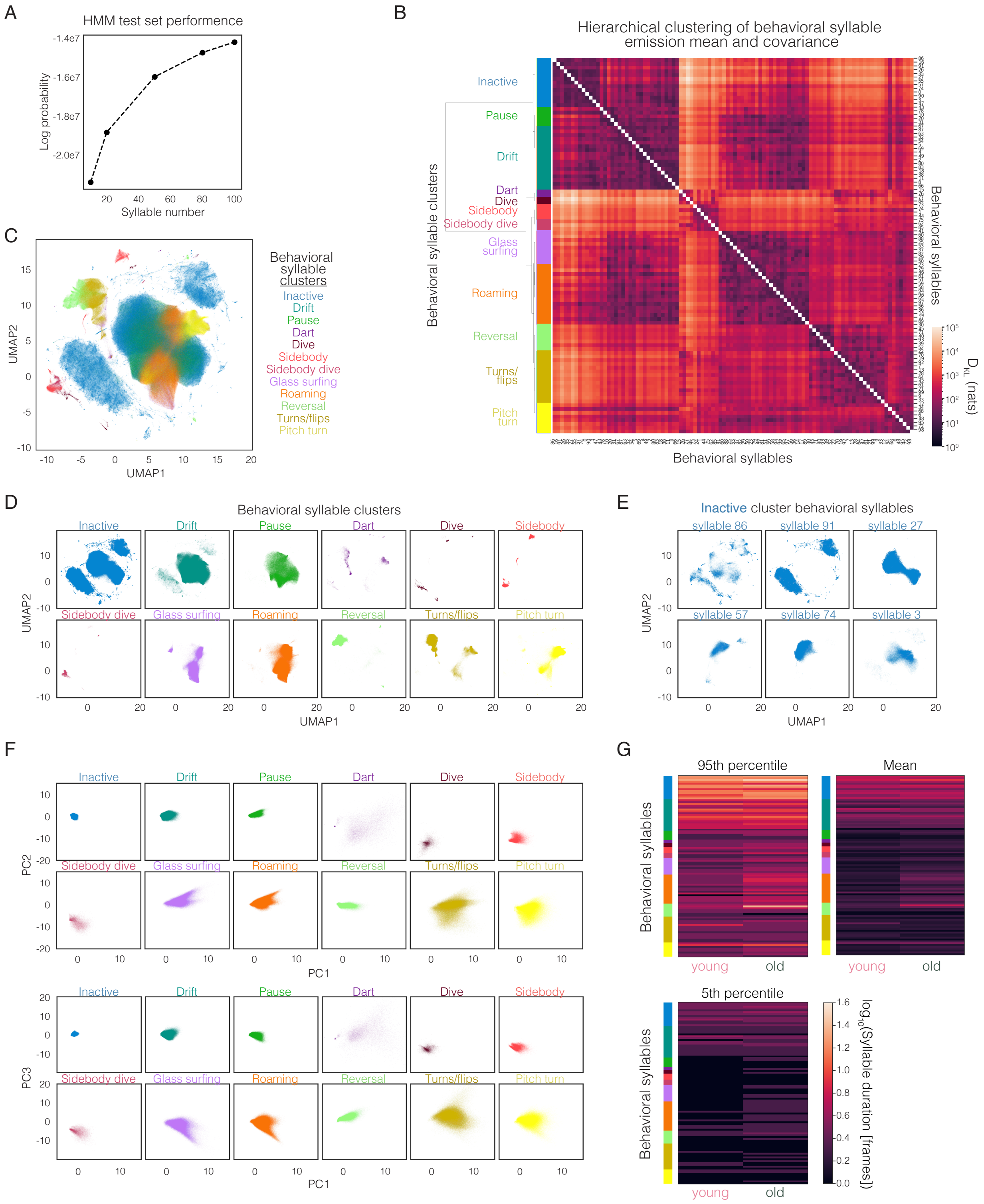

Fig. S4. HMM optimization and HMM-derived behavioral syllable clustering. (**A**) Cross-validation log probability of different HMM models with varying numbers of behavioral syllables using stochastic EM fitting procedure. (**B**) Hierarchical clustering of behavioral syllables using symmetrized D_{KL} of HMM emission distributions with the resulting behavioral syllable clusters shown in color along left. (**C**) UMAP embedding of pose features PCs of data

from a single day for eight different fish (five young and four old) down-sampled from 20 Hz to 4 Hz frame rate, with each scatter point representing the embedding of a single frame of recording and color maps indicating the behavioral syllable cluster. (**D**) Same as (**C**) but frames from distinct behavioral syllable clusters are plotted separately. (**E**) Same as (**C**) but only plotting frames from distinct behavioral syllables within the "inactive" behavioral syllable cluster. (**F**) Top three pose feature PCs (PC1, PC2, PC3) with color maps indicating the behavioral syllable cluster and distinct behavioral syllable clusters plotted separately. (**G**) Mean, 5th percentile, and 95th percentile syllable duration (frames) comparing young (45-day old) and old (270-day old) animals from a single day for nine different fish (five young and four old).

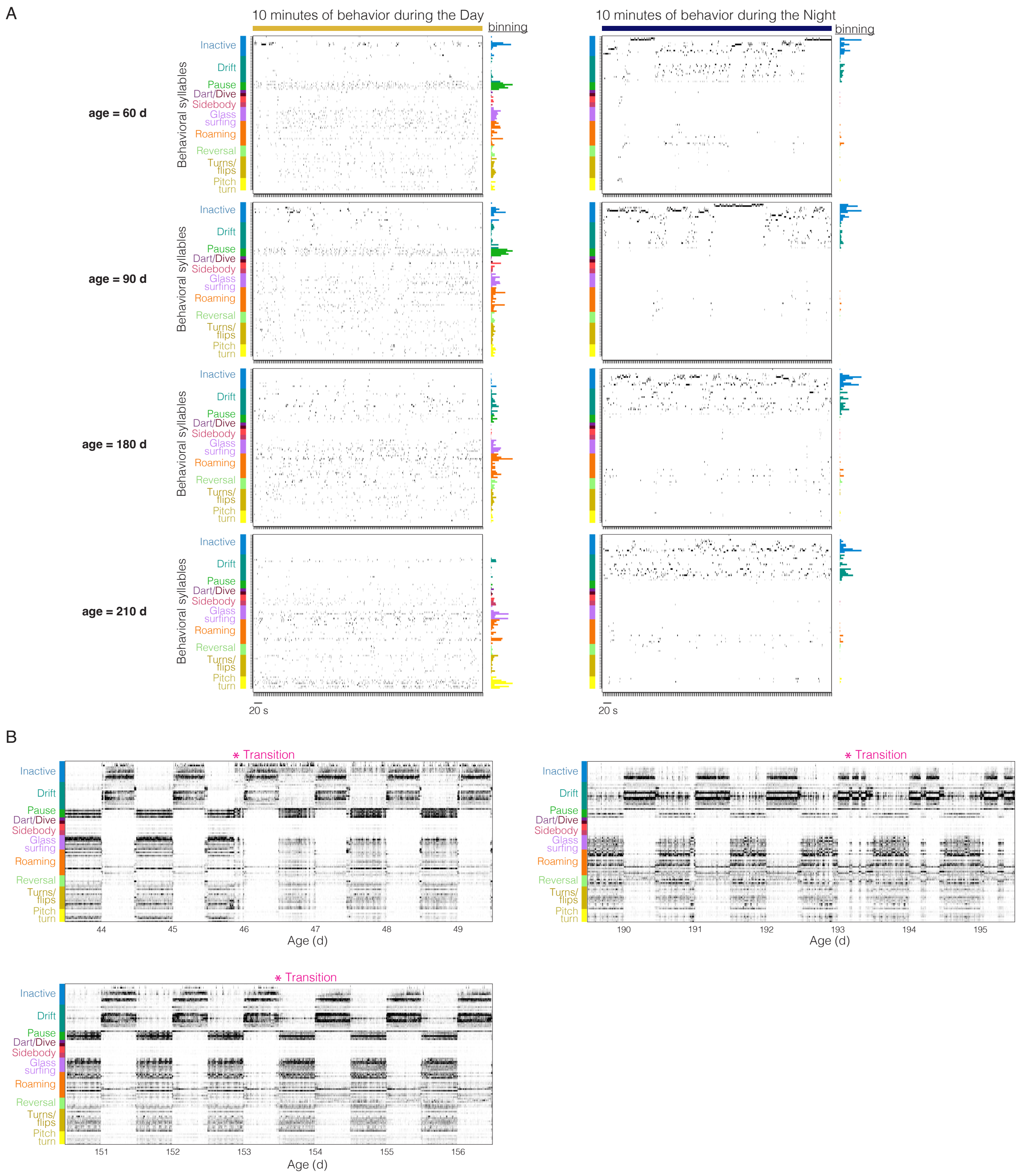

Fig. S5. Behavioral syllable dynamics at different ages and during transitions. (**A**) Sampling of 100 HMM-derived behavioral syllables for a single fish at four different ages during 10-min interval during the day (left) and 10-min interval during the night (right). Throughout figure, behavioral syllables are ordered according to similarity and hierarchical clustering of distinct types of behavior shown in color along left. Behavioral syllables are ordered as in Fig. 1I. Histogram shows time spent in each behavioral syllable during the 10-min bin on right. (**B**) Behavioral syllable usage for a single animal for windows of life during a transition highlighted in Fig. 1K.

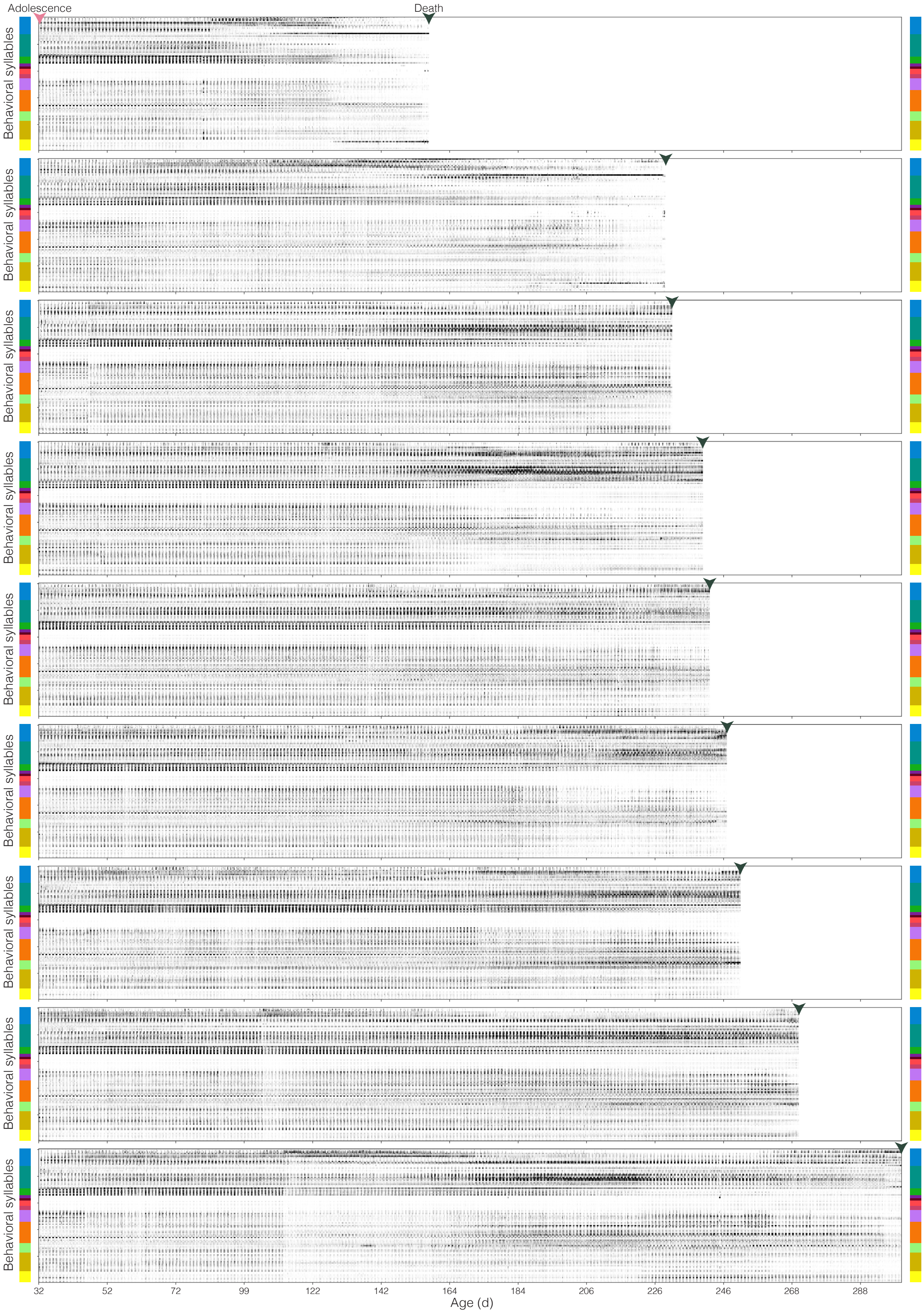

Adolescence
Death
Behavioral syllables
32
52
72
99
122
142
164
184
206
226
246
268
288
Age (d)

Fig. S6. Whole-lifespan behavioral syllable use for individual animals from adolescence until death. Animals ordered based on lifespan from shortest to longest lived. Behavioral syllables are ordered according to similarity and hierarchical clustering of distinct types of behavior shown in color along left and right. Behavioral syllables are ordered as in Fig. 1I.

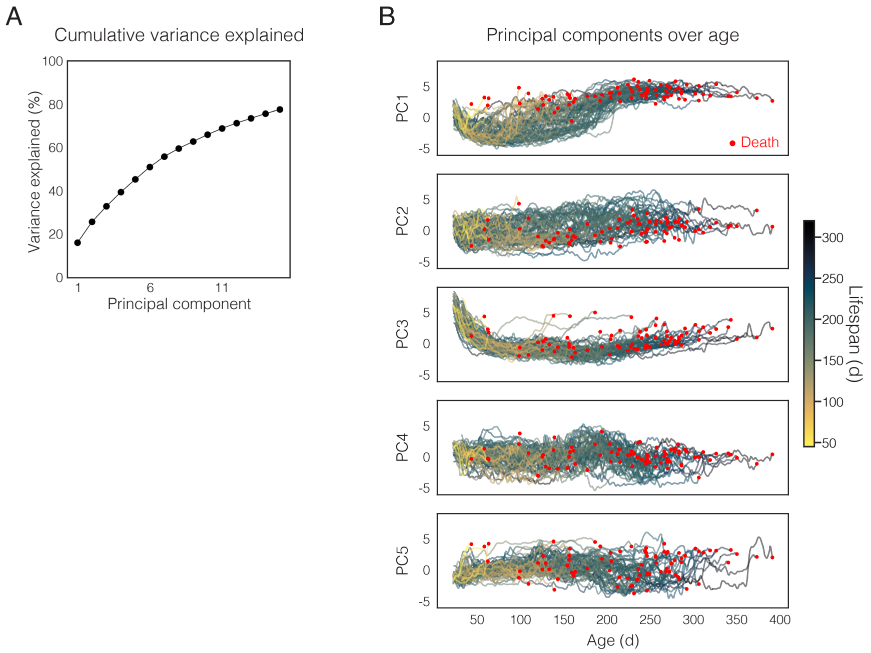

Fig. S7. PCA of 45 tensor components. (**A**) Cumulative variance explained for increasing principal components. The top three PCs are used in visualizations in Fig. 2E-G and Fig. 3B,H. (**B**) Top five PCs plotted across age of 81 animals colored by future lifespan. Age of death indicated by red circle for each animal.

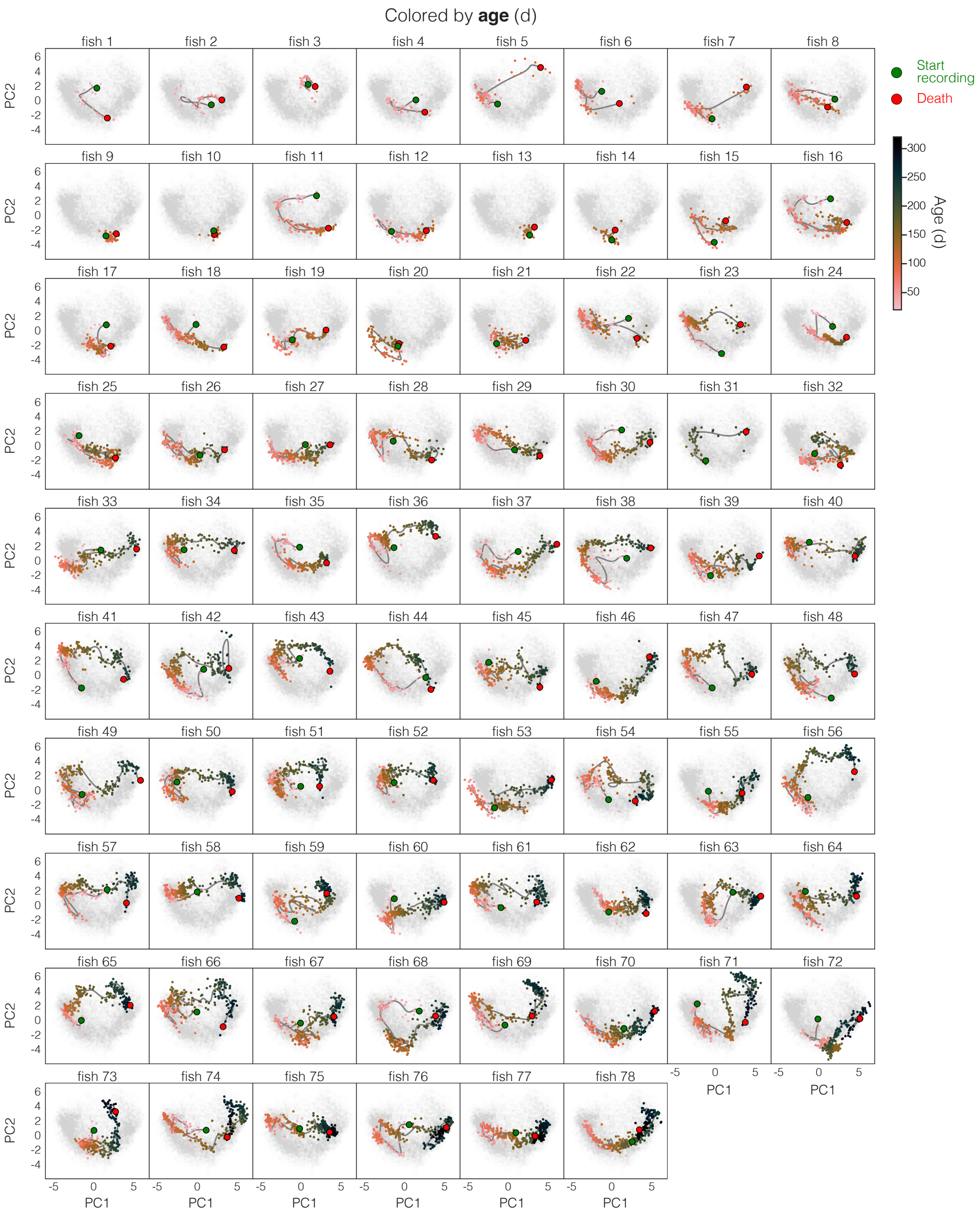

Fig. S8. Individual animal aging trajectories. Top two PCs (PC1 and PC2) of 45 TC data to visualize aging trajectories of 78 individual animals from start of recording (green circle) to death (red circle). Each animal is shown in a separate plot. Points show individual days. Line shows smoothed trajectory. Each point colored by animal age. The whole population is shown in light grey scatter points for reference.

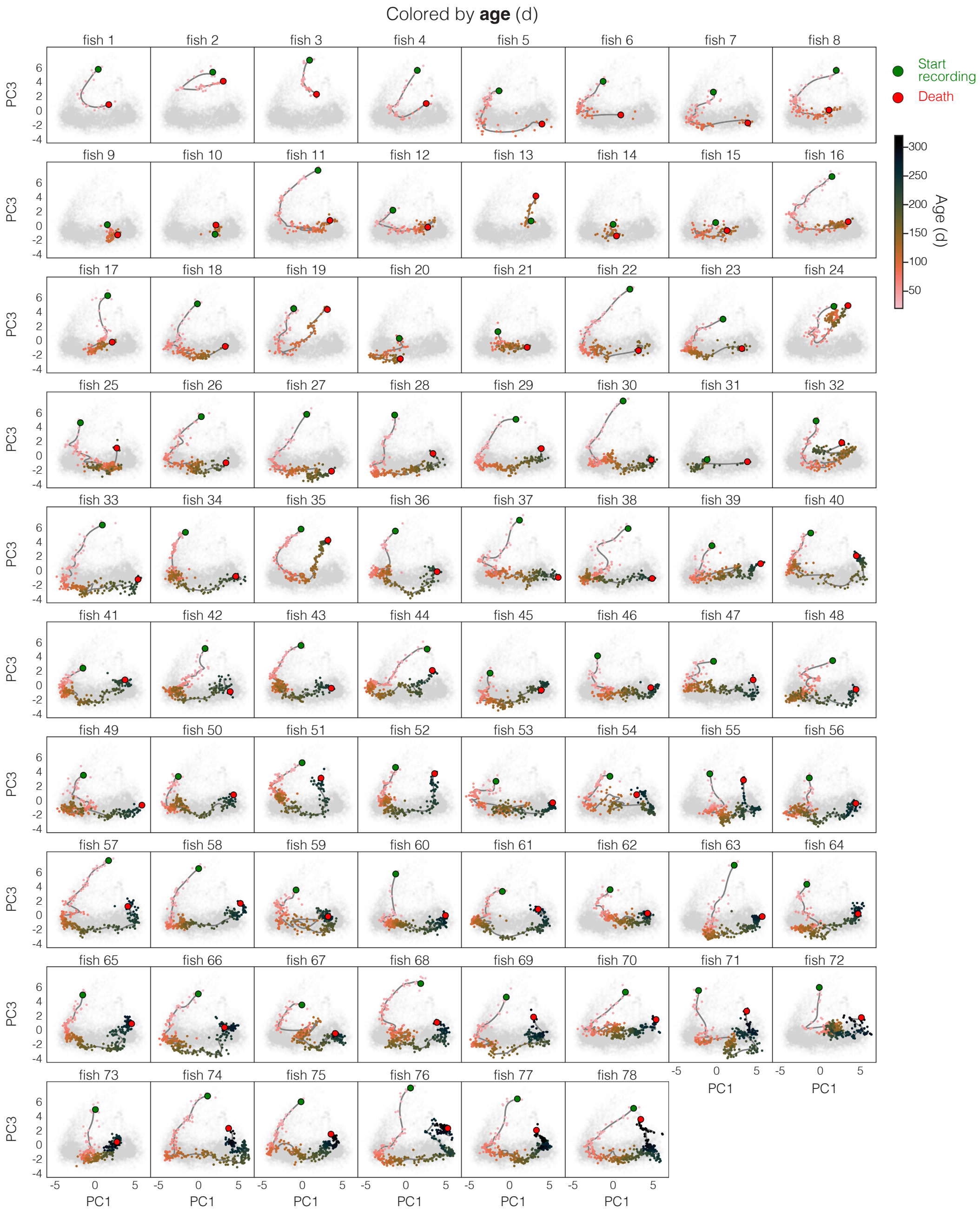

Fig. S9. Individual animal aging trajectories. Top three PCs (PC1 and PC3) of 45 TC data to visualize aging trajectories of 78 individual animals from start of recording (green circle) to death (red circle). Each animal is shown in a separate plot. Points show individual days. Line shows smoothed trajectory. Each point colored by animal age. The whole population is shown in light grey scatter points for reference.

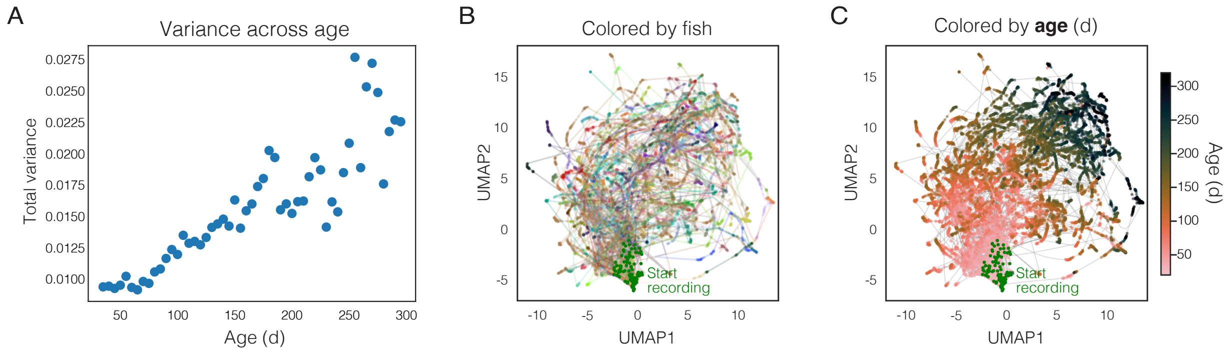

Fig. S10. Aging trajectories across life. (**A**) Summed variance of all 45 TCs at a range of ages across life. (**B**-**C**) UMAP embedding of 45 TC data to visualize aging trajectories. Points show individual days. Line shows smoothed trajectory. With each animal in a different color (**B**) or with each point colored by animal age (**C**).

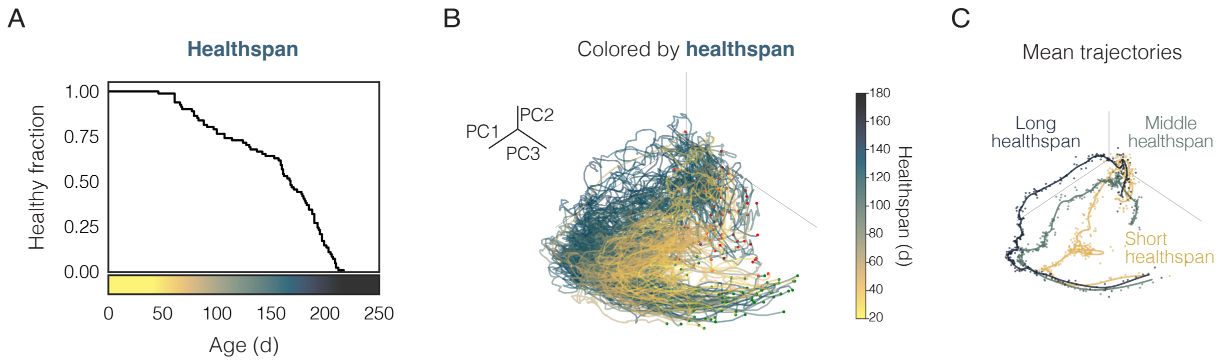

Fig. S11. Behavior-based aging trajectories of short-healthspan vs. long-healthspan animals differ. (**A**) Kaplan–Meier healthspan curve of tracked animals (n=81). (**B**) PCA aging trajectories of 81 animals colored by healthspan (i.e., healthy life), defined for killifish as remaining active and consuming all available food. (**C**) Mean trajectory of distinct healthspan groups (short: 25th percentile; middle: 25-50th percentile; long: 75th percentile).

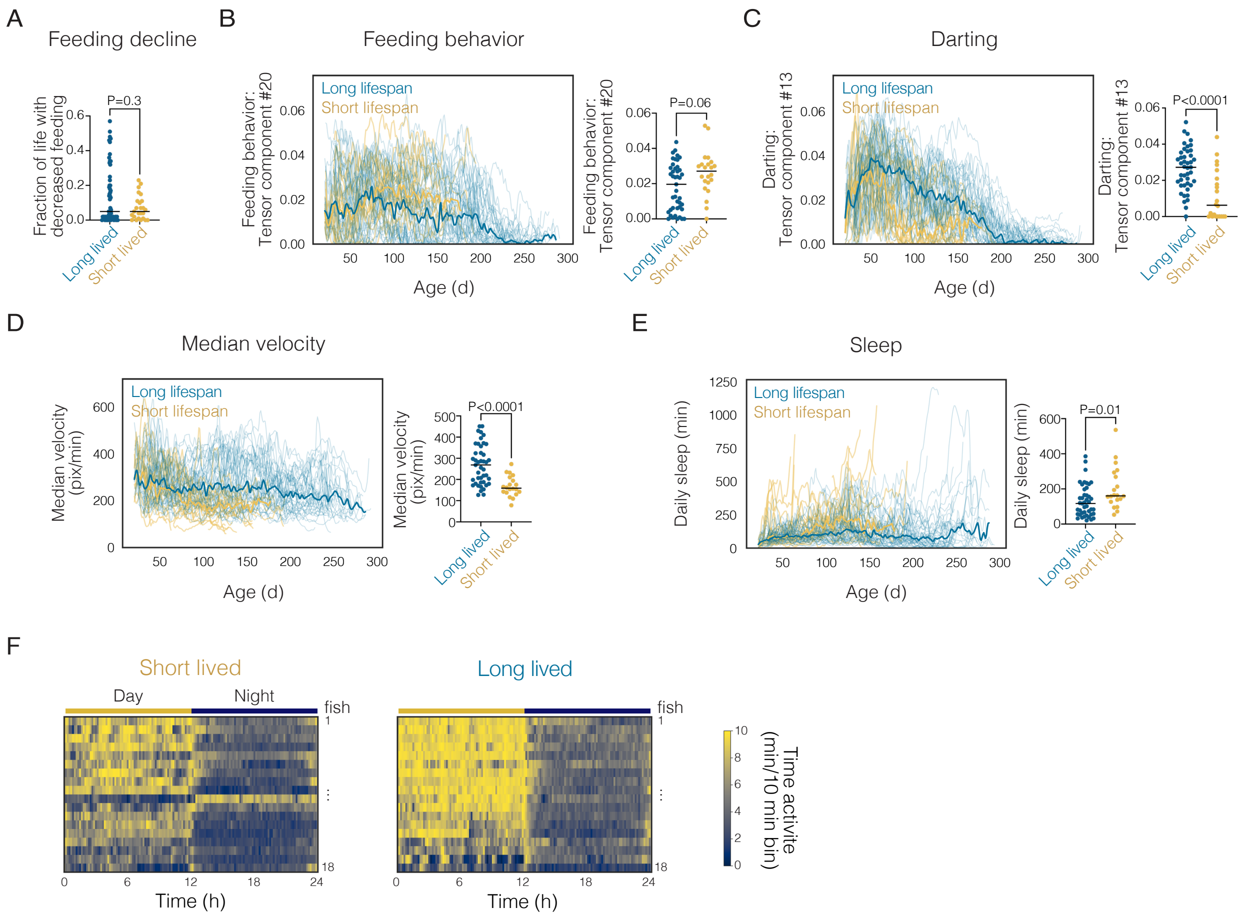

Fig. S12. Behavior and locomotor activity of long- vs. short-lived animals. (**A**) Proportion of life prior to death with reduced food consumption resulting in residual uneaten food. Feeding behavior (weighting of tensor component #20 in Fig. 2D) (**B**), vigorous darting behavior (weighting of tensor component #13 in Fig. 2D) (**C**), median velocity (**D**), and total daily sleep (**E**) across life (left) and at 100 days old (right) grouped by lifespan. Mann Whitney test for significances. (**F**) Heatmap of time spent active across 24 hr at 100 days old with each row representing a different animal and animals grouped by lifespan. Light/dark cycle indicated by yellow/blue bar.

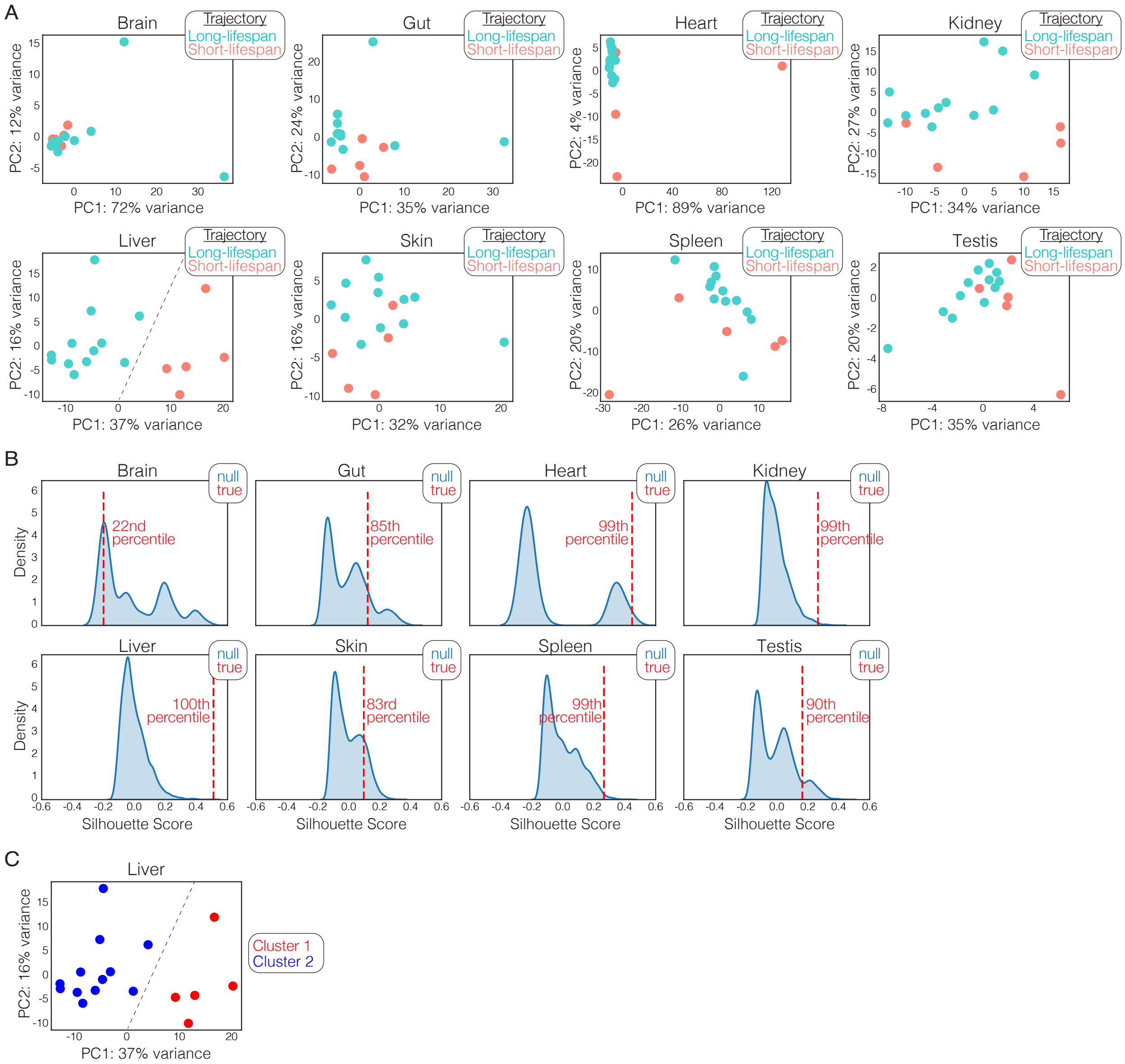

Fig. S13. Multi-organ transcriptomics. (**A**) PCA of whole transcriptome for each animal in the middle-aged group (150 d; n=17) colored by future-lifespan trajectory based on behavior. Each plot is a different organ. (**B**) Silhouette score in PC1/PC2 space of true labels (long-lifespan vs short-lifespan trajectory based on behavior) indicated by red dashed line and calculated percentile compared to null distribution build by permutations of randomized group assignment across all n=17 samples (blue distribution). (**C**) Unbiased k-means clustering of the liver PC1/PC2 data into two clusters results in perfect match of the observed short-lifespan and long-lifespan trajectory based on behavior labels shown in (**A**).

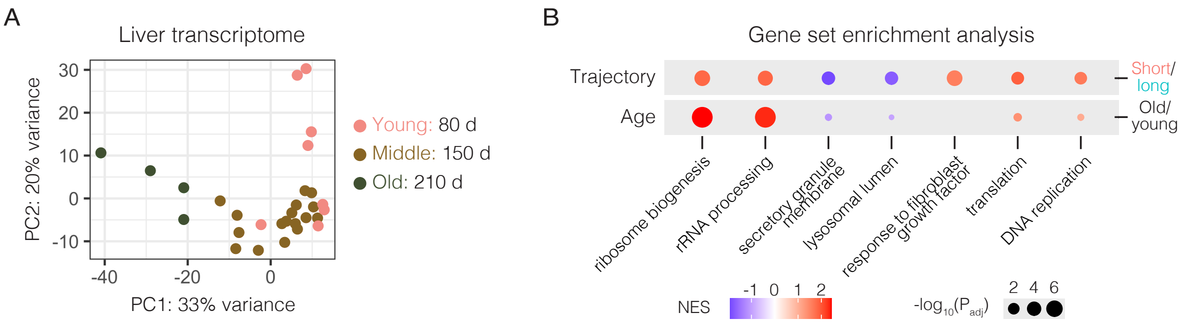

Fig. S14. Liver transcriptome is different both with age and lifespan trajectory. (**A**) PCA of whole transcriptome for each animal combining the young (80 d; n=8), middle-aged (150 d; n=17), and old (≥210 d, n=4) cohorts colored age. (**B**) Gene set enrichment analysis of the liver transcriptome as a function of lifespan trajectories based on behavior (short- vs. long-lifespan trajectory) compared to age (old vs. young) highlighting significant Gene Ontology (GO) terms. NES, normalized enrichment score. P_{adj}, P-value adjusted for multiple hypothesis testing.

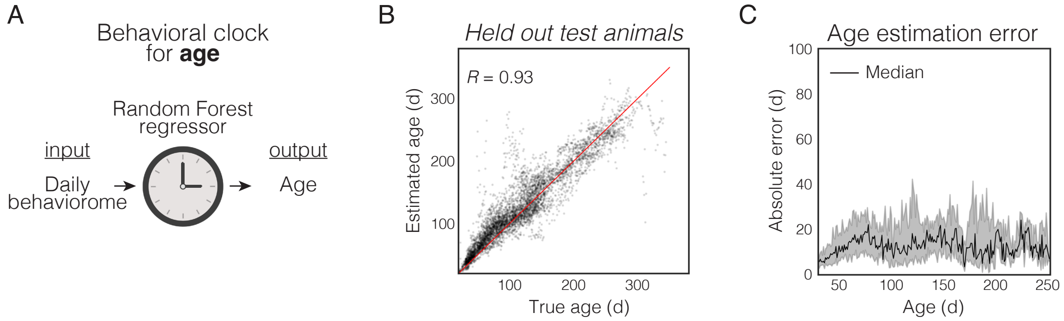

Fig. S15. Behavioral clock for age. (**A**) Random forest regression models trained on behavioral TC data to estimate age (i.e., behavioral clock for age). (**B**) Held out test set cohorts (36 held-out animals) model estimated age vs. true age. (**C**) Held out test set cohorts (36 held-out animals) model age estimation absolute error calculated across age.

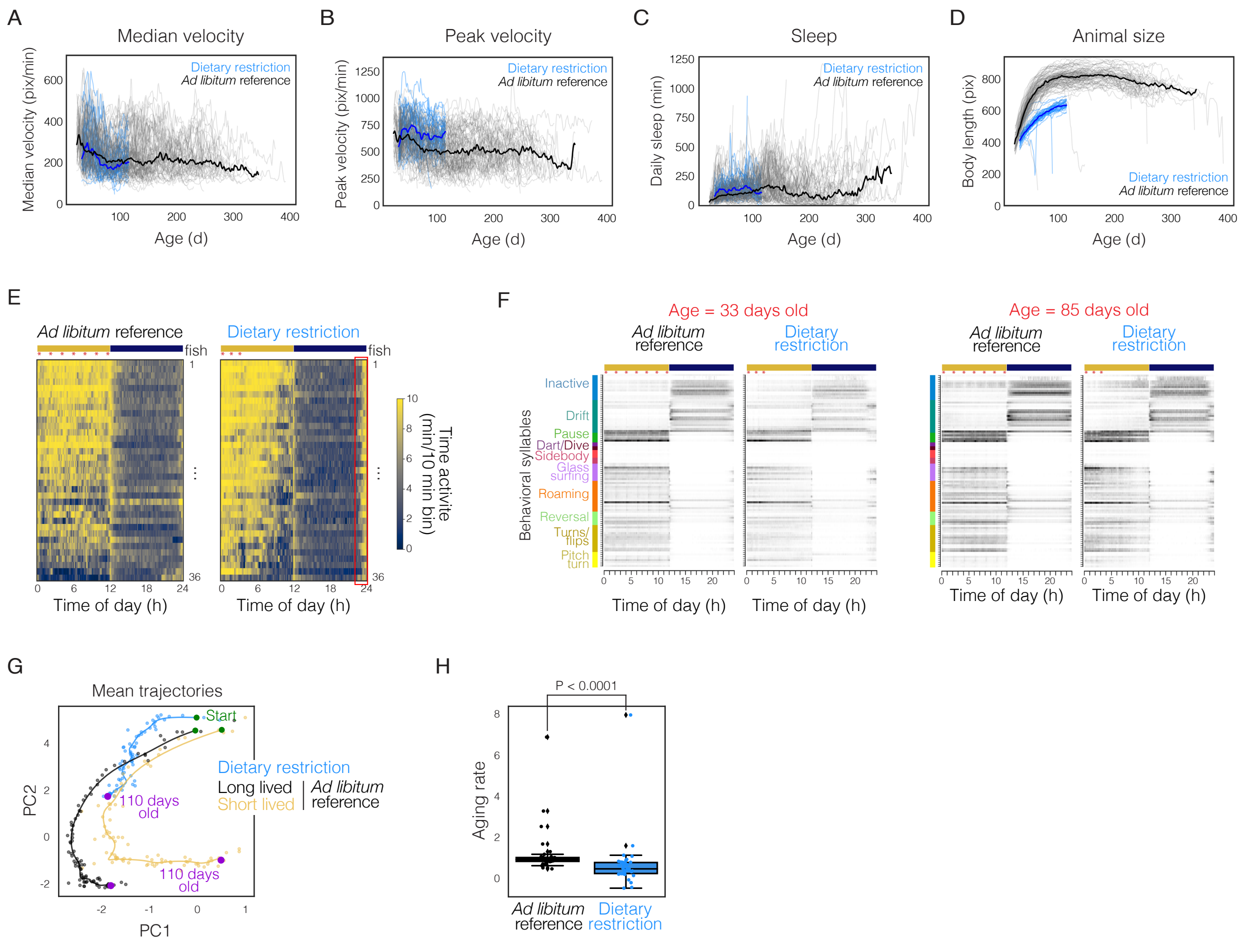

Fig. S16. Behavior, locomotor activity, and size with dietary restricted feeding. Median velocity (**A**), peak velocity (**B**), total daily sleep (**C**), and animal size (**D**) across recordings comparing animals under dietary restriction (n=39; recorded until 110 d old) to *ad libitum* reference (n=80; recorded full lifespan). (**E**) Heatmap of time spent active across 24 hr at 54 d old with each row representing a different animal. Animals under dietary restriction (right; n=36) are compared to randomly selected *ad libitum* reference animals from previous cohorts (left; n=36). Red asterisks indicate feeding times. Light/dark cycle is indicated by yellow/blue bar. Red rectangle underscores elevated activity before the lights turn on for the animals under dietary restriction. (**F**) Mean behavioral syllable usage across 24 hr for all animals under dietary restriction (right; n=39) and all animals from the *ad libitum* reference cohorts (left; n=80) at 33 d old and 85 d old. Behavioral syllables are ordered as in Fig. 1I. (**G**) Mean behavioral aging trajectory of long-lived *ad libitum* reference animals (black; lifespan > 200 days); short-lived *ad libitum* reference animals (yellow; lifespan<200 days); vs. dietary restriction animals (blue). Purple points indicate when animals reach 110 days old. By 110 days, the dietary restriction animals are behaviorally more similar to ~50-day old *ad libitum* animals. However, it is not possible to determine if the dietary restricted animals follow an *ad libitum* short-lived or long-lived trajectory because at this point in the trajectory, there is not yet a robust difference between the behavior of short-lived and long-lived animals (Fig. 3B,D-E; Fig. 4F-G,I). (**H**) Aging rate (i.e., the slope of behavioral clock estimated age vs true age) of *ad libitum* reference animals (black) vs. dietary restriction animals (blue).

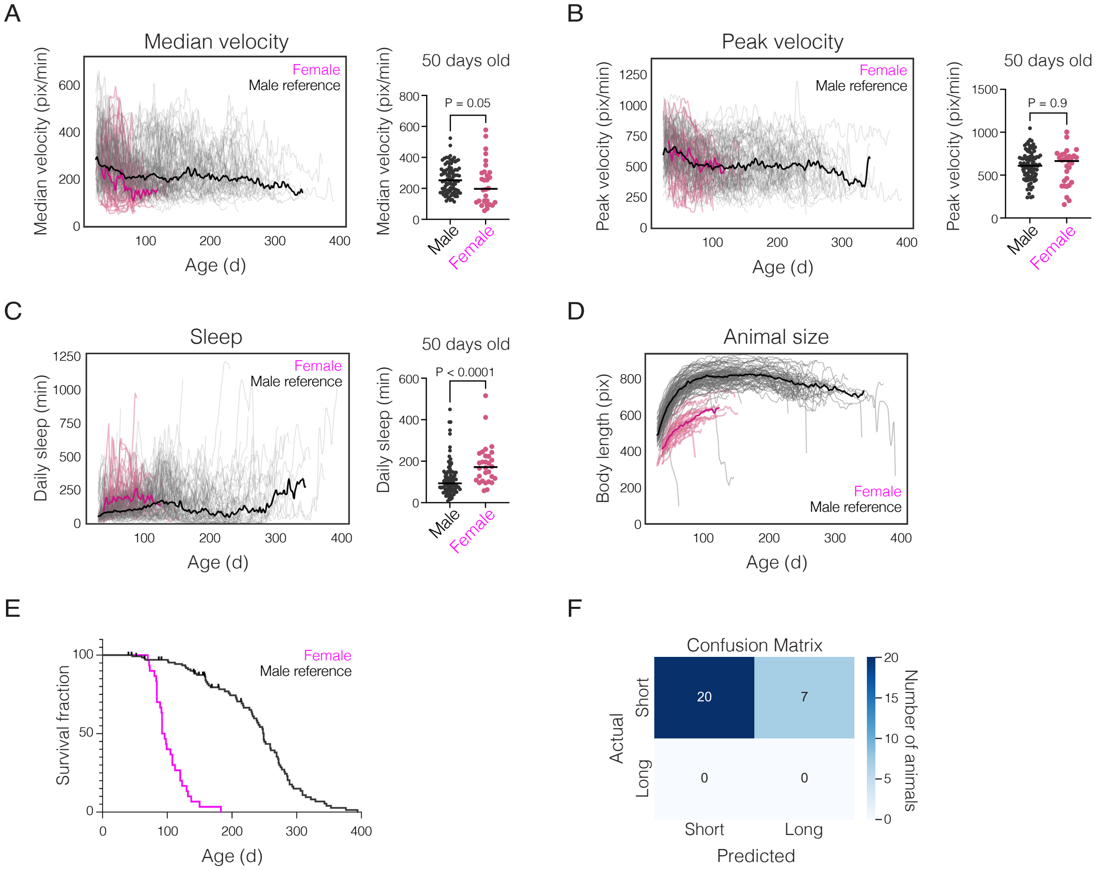

Fig. S17. Behavior, locomotor activity, size and lifespan of females. Median velocity (**A**), peak velocity (**B**), and total daily sleep (**C**) across life (left) and at 50 days old (right) comparing females to the male reference. Mann Whitney test for significance. (**D**) Animal size across life. (**E**) Kaplan–Meier lifespan curve of female animals (n=31) compared to male reference (n=118). (**F**) Confusion matrix for random forest classification model predictions on the female cohort (n=27) based on behavior at 70 days old. Animals are classified as either short- or long-lived (i.e., behavioral classifier for lifespan).

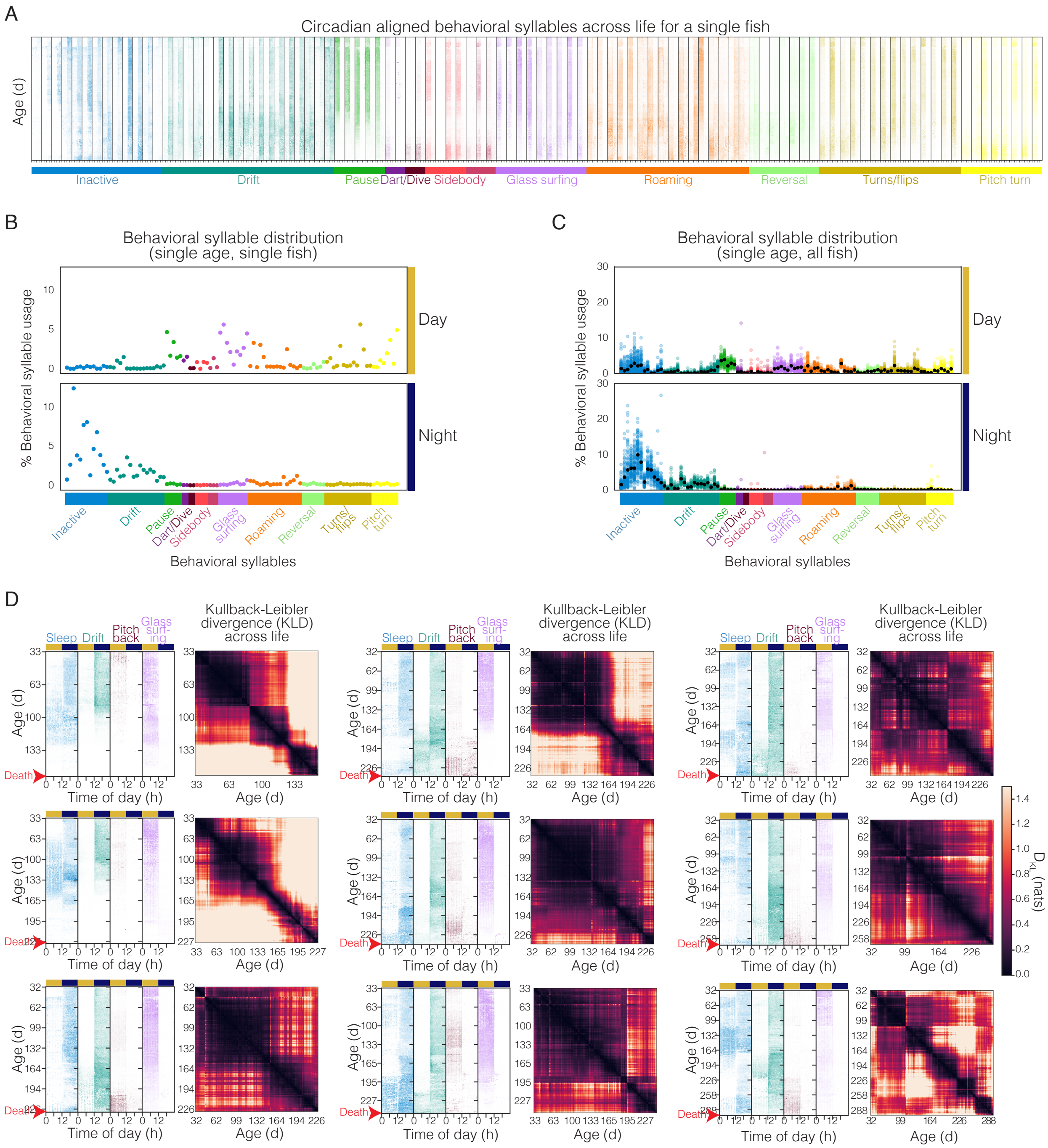

Fig. S18. Changes in whole-lifespan behavioral syllable use for individual animals. (**A**) Circadian-aligned, whole lifespan heatmaps for all 100 behavioral syllables of example animal in Fig. 6A-D. (**B**) Behavioral syllable usage distribution for day and night of a single animal at a single age. (**C**) Behavioral syllable usage distribution for day and night of all tracked animal at a single age. (**D**) Circadian-aligned, whole-lifespan heatmaps of four select behaviors (as in Fig. 6B) for nine individual animals (same animals shown in fig. S6) aligned with cross-correlation of symmetrized D_{KL} of the behavioral syllable usage distribution comparing all days in one animal's life to all other days ordered from youth (32 d) to death. Light/dark cycle is indicated by yellow/blue bar.

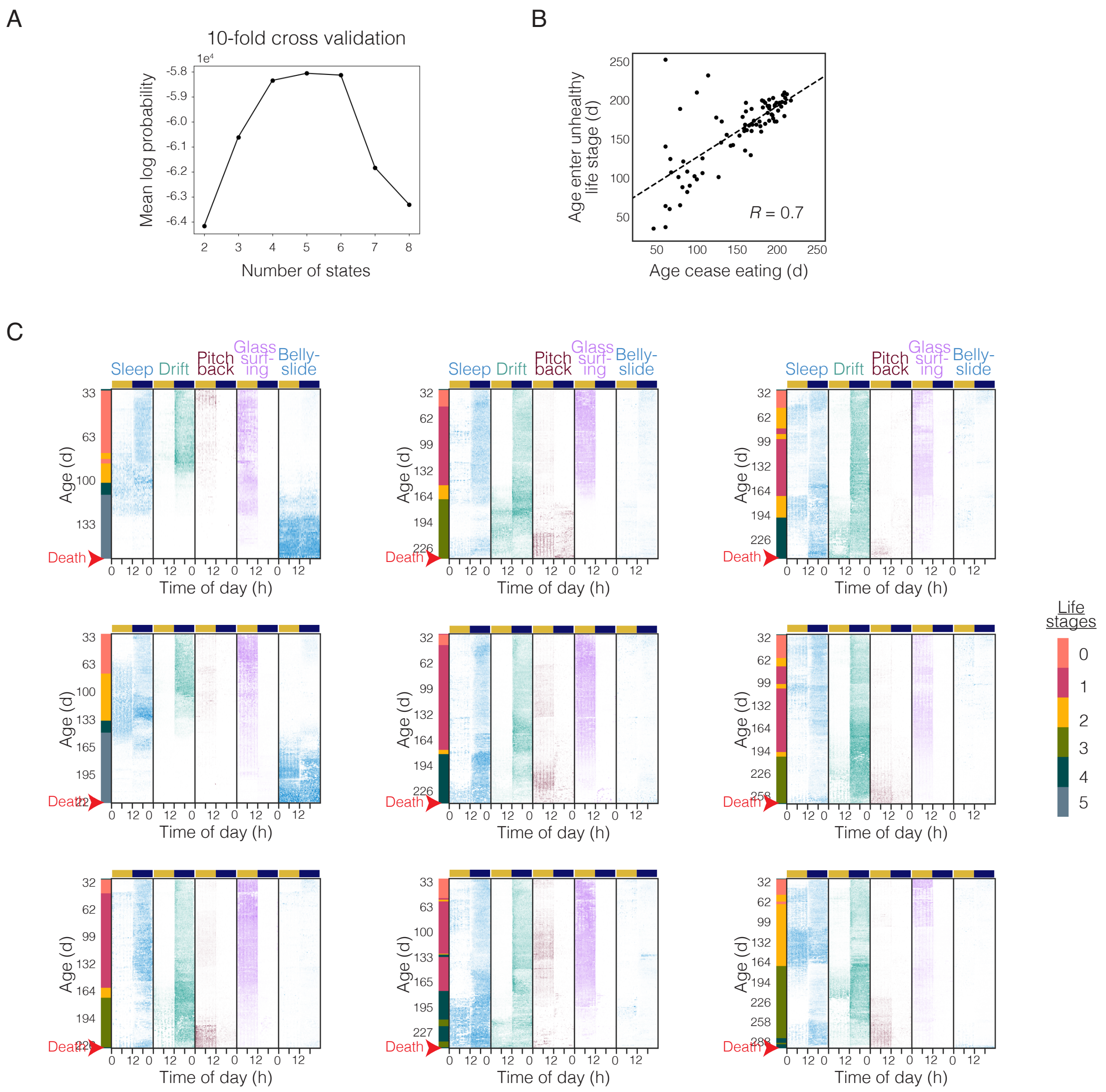

Fig. S19. HMM life stage model optimization and output. (**A**) Mean log probability of 10-fold cross validation varying the number of states. (**B**) Correlation of age at which animals cease eating, with the youngest age an animal enters either unhealthy (green or blue) life stage. (**C**) Circadian-aligned, whole-lifespan heatmaps of five select behaviors for nine individual animals (same animals shown in fig. S6 and fig. S18) aligned with HMM-predicted life stages along the left. Light/dark cycle is indicated by yellow/blue bar.

Movie S1.
Example movie from one recording table of 12 male killifish with cameras mounted overhead with infrared backlighting (20 frames per second).

Movie S2.
Deep convolutional neural network prediction of key points along the body – snout, midbody, endbody, tail, fan, and sidebody for example Movie S1.

Data S1. (separate file)
Fifty-seven features describing pose feature dynamics across time calculated by either the mean and/or standard deviation (depending on the feature) of pose features (evaluated from the x-/y-coordinates of tracked key points) over a 10-frame rolling window.

Data S2. (separate file)
Liver transcriptomics comparison of short-lifespan vs long-lifespan trajectory groups at 150 days old (n=17) shown in Fig. 3G-K. (**Barcodes**) Barcode and detailed sample information. (**DEseq sheet**) Differential expression analysis results with log2FoldChange>0 if gene expression is elevated in the short-lifespan trajectory group relative to the long-lifespan trajectory group. (**PEA sheet**) Over-representation analysis for significantly differentially expressed genes between short-lived vs. long-lived trajectory using Gene Ontology (GO) enrichment analysis. GO pathways are up (positive charge) if driven by genes significantly elevated in the short-lifespan trajectory group relative to the long-lifespan trajectory group. (**GSEA sheet**) Gene set enrichment analysis results with positive enrichment score if elevated in the short-lifespan trajectory group relative to the long-lifespan trajectory group. (**TopGenes sheet**) Differentially expressed genes ($P_{adj} > 0.1$) between the short-lifespan vs long-lifespan trajectory groups at 150 days old grouped by pathways. Focusing on pathways elevated in the short-lifespan trajectory group relative to the long-lifespan trajectory group.
